# Cytochalasans and Their Impact on Actin Filament Remodeling

**DOI:** 10.3390/biom13081247

**Published:** 2023-08-15

**Authors:** Christopher Lambert, Katharina Schmidt, Marius Karger, Marc Stadler, Theresia E. B. Stradal, Klemens Rottner

**Affiliations:** 1 Molecular Cell Biology Group, Helmholtz Centre for Infection Research (HZI), Inhoffenstrasse 7, 38124 Braunschweig, Germany; 2 Department of Cell Biology, Helmholtz Centre for Infection Research (HZI), Inhoffenstrasse 7, 38124 Braunschweig, Germany; 3 Department of Microbial Drugs, Helmholtz Centre for Infection Research and German Centre for Infection Research (DZIF), Partner Site Hannover/Braunschweig, Inhoffenstrasse 7, 38124 Braunschweig, Germany; marc.stadler@helmholtz-hzi.de; 4 Division of Molecular Cell Biology, Zoological Institute, Technische Universität Braunschweig, Spielmannstrasse 7, 38106 Braunschweig, Germany; 5 Braunschweig Integrated Centre of Systems Biology (BRICS), 38106 Braunschweig, Germany

**Keywords:** eukaryotic actin cytoskeleton, actin inhibitors, structure–activity relationship, actin binding proteins, secondary metabolites, chemo-diversity

## Abstract

The eukaryotic actin cytoskeleton comprises the protein itself in its monomeric and filamentous forms, G- and F-actin, as well as multiple interaction partners (actin-binding proteins, ABPs). This gives rise to a temporally and spatially controlled, dynamic network, eliciting a plethora of motility-associated processes. To interfere with the complex inter- and intracellular interactions the actin cytoskeleton confers, small molecular inhibitors have been used, foremost of all to study the relevance of actin filaments and their turnover for various cellular processes. The most prominent inhibitors act by, e.g., sequestering monomers or by interfering with the polymerization of new filaments and the elongation of existing filaments. Among these inhibitors used as tool compounds are the cytochalasans, fungal secondary metabolites known for decades and exploited for their F-actin polymerization inhibitory capabilities. In spite of their application as tool compounds for decades, comprehensive data are lacking that explain (i) how the structural deviances of the more than 400 cytochalasans described to date influence their bioactivity mechanistically and (ii) how the intricate network of ABPs reacts (or adapts) to cytochalasan binding. This review thus aims to summarize the information available concerning the structural features of cytochalasans and their influence on the described activities on cell morphology and actin cytoskeleton organization in eukaryotic cells.

## 1. Introduction

**Figure 1:**
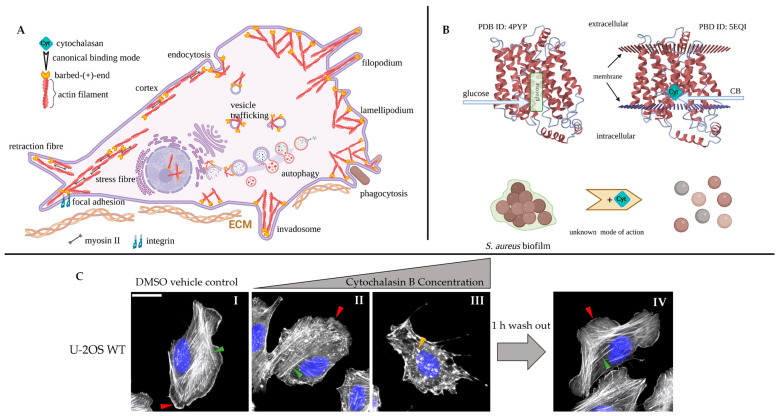
(**A**). Schematized overview of selected, intracellular actin filament structures and the cellular processes these structures contribute to (filaments and molecules not drawn to scale). The respective positions of filament barbed ends, which cytochalasans are assumed to bind to, are also indicated. Although all actin filament barbed ends are potential targets of cytochalasans, in principle, differential effects on different actin structures can be observed, but the precise molecular reasons for this have yet to be established (for details, see text). (**B**) Selected, actin-independent activities of cytochalasans. (**C**). Representative epifluorescence images of human osteosarcoma cells stained for nuclear DNA using DAPI (pseudocolored in blue) and actin filaments with fluorescently-coupled phalloidin (in grey), treated with vehicle control (DMSO, **I**) or low and high concentrations of CB (**1, II + III**) for 1 h, with the latter followed by 1 h washout, as indicated (**IV**). Control U-2 OS cells display discernible stress fibers (**I**, green arrowhead) and F-actin-rich lamellipodia (**I**, red arrowhead). Low-dose CB (**1**) treatments remove lamellipodia at the cell periphery (**II**, red arrowhead) and cause partial induction of F-actin-rich spots, the precise nature of which is elusive, with stress fibers remaining largely intact (**II**, green arrowhead). The latter disappear upon high-dose CB (**1**); aggregates grow even larger (**III**, orange arrowhead). In spite of these drastic phenotypic changes, a washout for 1 h is sufficient for full recovery of the U-2 OS cell actin network (**IV**, red arrowhead: lamellipodium, green arrowhead: stress fiber). Figure subpanels (**A**,**B**) were created with BioRender.com.

Fungi are capable of producing an astonishing diversity of bioactive secondary metabolites, which are dispensable for survival in contrast to primary metabolites but are usually thought to serve as fitness factors improving competitiveness in response to challenges in their natural environments [1]. Among those secondary metabolites are cytochalasans, small-molecule bioactive hybrid compounds synthesized in concerted action by a polyketide synthase and a non-ribosomal peptide synthetase (PKS-NRPS) [2]. They are well known to occur throughout different taxonomic orders, such as the Xylariales, Sordariales, and Diaporthales, amongst others, in the phylum of the Ascomycota [3,4,5]. Over the past decades, an astonishing arsenal of cytochalasan diversity has been described, with over 400 structures of natural origin elucidated to date [6]. After their first description and isolation in 1966 [7], the scientific community of cell biologists and natural product chemists alike quickly grew interested in their bioactivity due to their eponymous influence on cell shape and behavior (“cyto” = cell; “chalasis” = slackening/relaxing). This relaxation is reflected in the well-known arborization and shriveling of normally well-spread, adherent cells upon high-dose cytochalasan administration. However, the range of biological effects further included the appearance of large, multi-nucleated cells after treatment of tissue culture cells, a phenomenon that was later linked with the inhibition of contractile ring constriction mediating cytokinesis [8]. This, for the first time, demonstrated the independence of this process from nuclear division, which later led to the recognition that the contractile ring consists of actin filaments and associated factors [9,10]. Most of the explorative work concerning the mode of action (MoA) of cytochalasans on actin, that is, its capability to bind to the fast-growing, so-called barbed (or plus-) ends of actin filaments (F-actin) and to effectively inhibit further addition of actin monomers [11,12], can be traced to the last millennium (reviewed by [13]). It should, however, not be neglected that cytochalasans were also shown to possess other interesting bioactivities apart from F-actin binding, best exemplified by the biofilm inhibitory capabilities exerted on prokaryotes [14] and the inhibitory effect on transporters such as the human glucose transporter 1 (hGLUT1; [15,16]), indicating a broader, understudied activity spectrum. Furthermore, actin in eukaryotes is involved in uncountable cellular functions, and whether or not actin is involved, cytochalasans have been shown to affect cells on multiple levels by disturbing (i) gene regulation and signaling cascades [17,18,19], (ii) several membrane channels and transporters [20,21,22,23,24], and (iii) phosphorylation patterns [23,25]. The detailed mechanisms behind these actions are not clear, but they might contribute to the cytochalasans constituting promising results against several pathogens and cancers. Indeed, selected cytochalasins were effective against several cancer cells and cancer types [19,26], to cite just a few. An extensive review and research about cyochalasans targeting different cancers was published by Trendowski [27,28] and Trendowski and colleagues [29]. Furthermore, in the context of microbial pathogens, multiple cytochalasans were found to be effective compounds against viruses, as they showed inhibitory capabilities for HIV proteases [30] and other HIV-related pathways [31,32,33], plus they slightly inhibited the macropinocytosis of SARS-CoV [34]. In addition, the observed activities against bacteria, viruses, parasites, and fungi, as well as the anti-inflammatory capabilities of cytochalasans, were nicely reviewed by Zhu et al. and Scherlach et al. [6,13]. Due to the broad activity spectrum of cytochalasans, further fields of applications, e.g., as pharmaceutical drugs in the clinical context [35], are discussed in the literature. However, prior to developing cytochalasans for broad-spectrum biotechnological application, a comprehensive and holistic understanding of their complex activities is necessary to be able to mitigate the natural risks associated with the application of an actin cytoskeletal inhibitor, potentially impacting various essential cell biological processes [6,36]. A broad overview of such processes, associated sites of activity, and exemplary images demonstrating the consequences for the intracellular filamentous actin network upon cytochalasan treatment are given in Figure 1.

The idea that cytochalasans interfere with F-actin polymerization at the barbed end still serves as the basis for their usage as model compounds to study F-actin-dependent effects [13]. However, the precise binding sites at the filament barbed ends of distinct cytochalasans and the consequences of these interactions for the various cellular barbed end interactors, and thus the specific regulation of actin assembly are far from clear. This is because the actin cytoskeleton of eukaryotic cells is tightly regulated by its binding partners, shaping actin architecture based on mechanical demands and feedback emerging from the cytoskeleton itself [37,38]. Comprehensive studies on how cytochalasans affect these regulatory processes are scarce. Additionally, although efforts have been made to report on the attempt to analyze the contributions of chemical modifications to the cytochalasins’ ability to bind actin, to date, no comprehensive structure–activity relationship (SAR) is available [2,6,13]. Considering how widely cytochalasans and all-for-one cytochalasin B (**1**) and D (**2**) (CB, **1** and CD, **2**) are used to probe for the relevance of actin dynamics in specific processes or to decipher the interplay between actin and its regulators, unraveling specific interactions and employing SAR studies will be instrumental for the correct interpretation of cytochalasan-caused effects imposed on the experimental model system of choice. This review thus aims to summarize reports and knowledge contextualizing the hitherto described chemodiversity, with its impact on actin, in particular its dynamics governed by ABPs and the network as a whole, to address open questions hampering a holistic, mechanistic understanding of cytochalasan mode of action.

## 2. Reports of Cytochalasans Impacting on the Morphology of Cellular Model Systems

**Figure 2:**
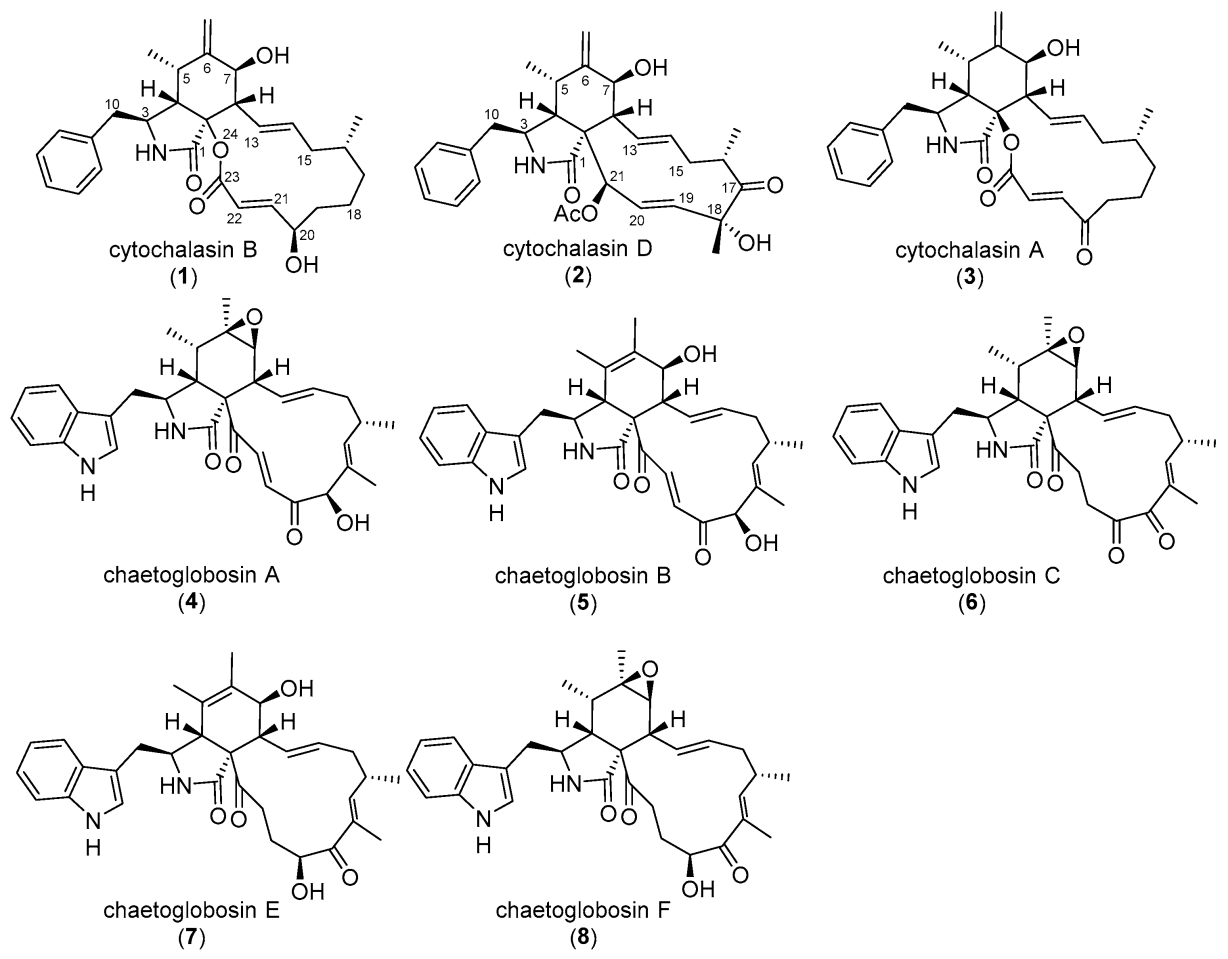
Structures of cytochalasin B (CB, **1**), D (CD, **2**), A (CA, **3**), and chaetoglobosin A-F (ChA-F, **4–8**).

The first descriptions of cytochalasin A (CA, **3**) and CB (**1**) by Rothweiler and Tamm (identical to dehydrophomin and phomin, respectively; independently isolated by [7]) date back to 1966 (Figure 2) [39]. Carter then described the effects of CB (**1**) on tissue culture cells, which effectively prevented cytoplasmic cleavage, leading to multinucleation, but also inhibited cellular motility and extruded nuclei after treatment [40]. This has later been corroborated by other authors [8,41,42]. In the following years, CB (**1**) was found to directly interact with actin but not other cytoskeletal microfilaments such as the tubulin network [9,43,44,45,46,47]. Peculiarly, the compound was shown to inhibit the growth of actin filaments [12,48,49,50], substantiating the phenomena described before [45]. Analogous effects were observed for the later isolated CD (**2**, Figure 2) [7], which included reports about a reduction in cell stiffness [51], however, with increased efficacy [52,53] of approximately ten times that of CB (**1**) [54]. The tryptophan-bearing cytochalasans, chaetoglobosin A-F (ChA-F, **4**–**8**; Figure 2), were also shown to lead to a severe increase in multinucleation in experimental models using tissue culture cells, suggesting similar mechanistic reasons behind cytochalasin- and chaetoglobosin-induced phenomena [55].

**Figure 3:**
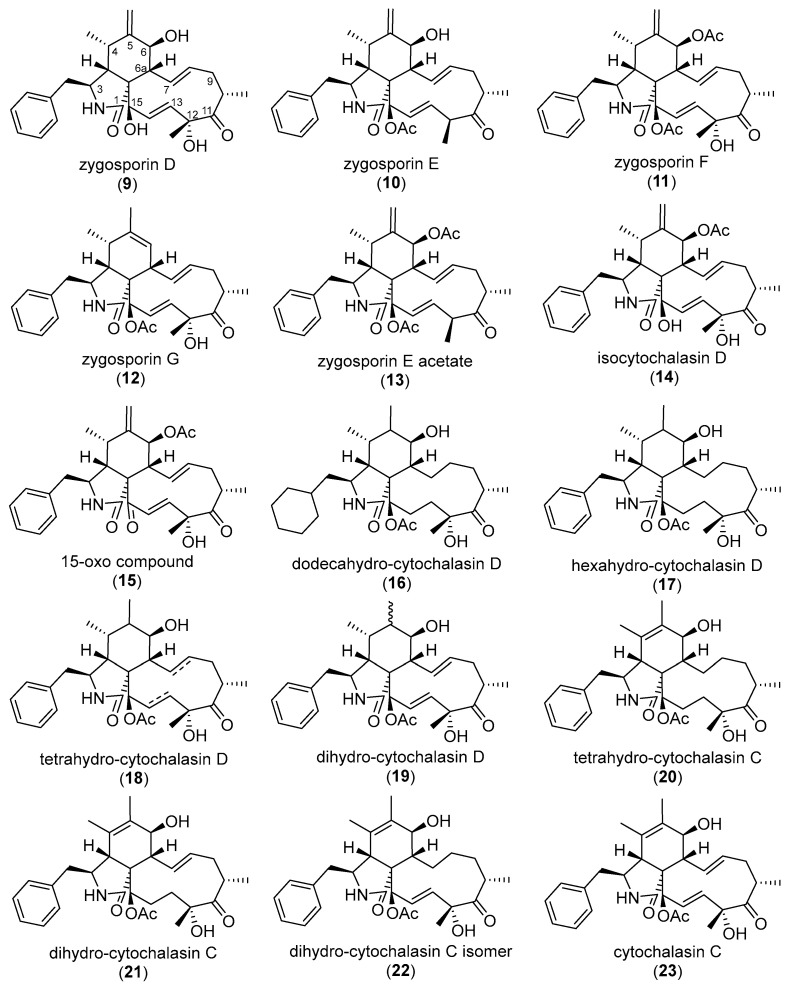

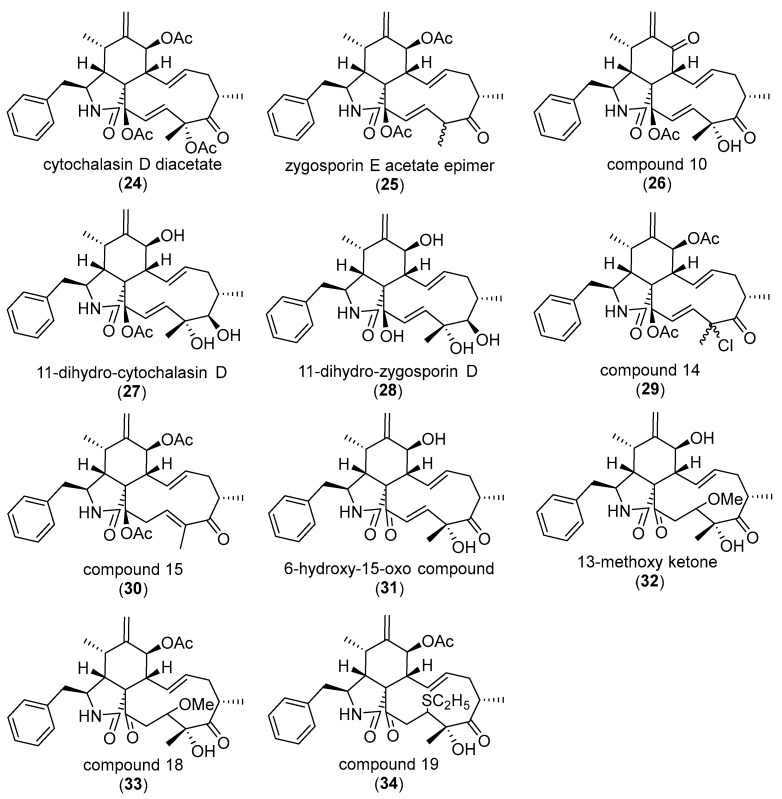

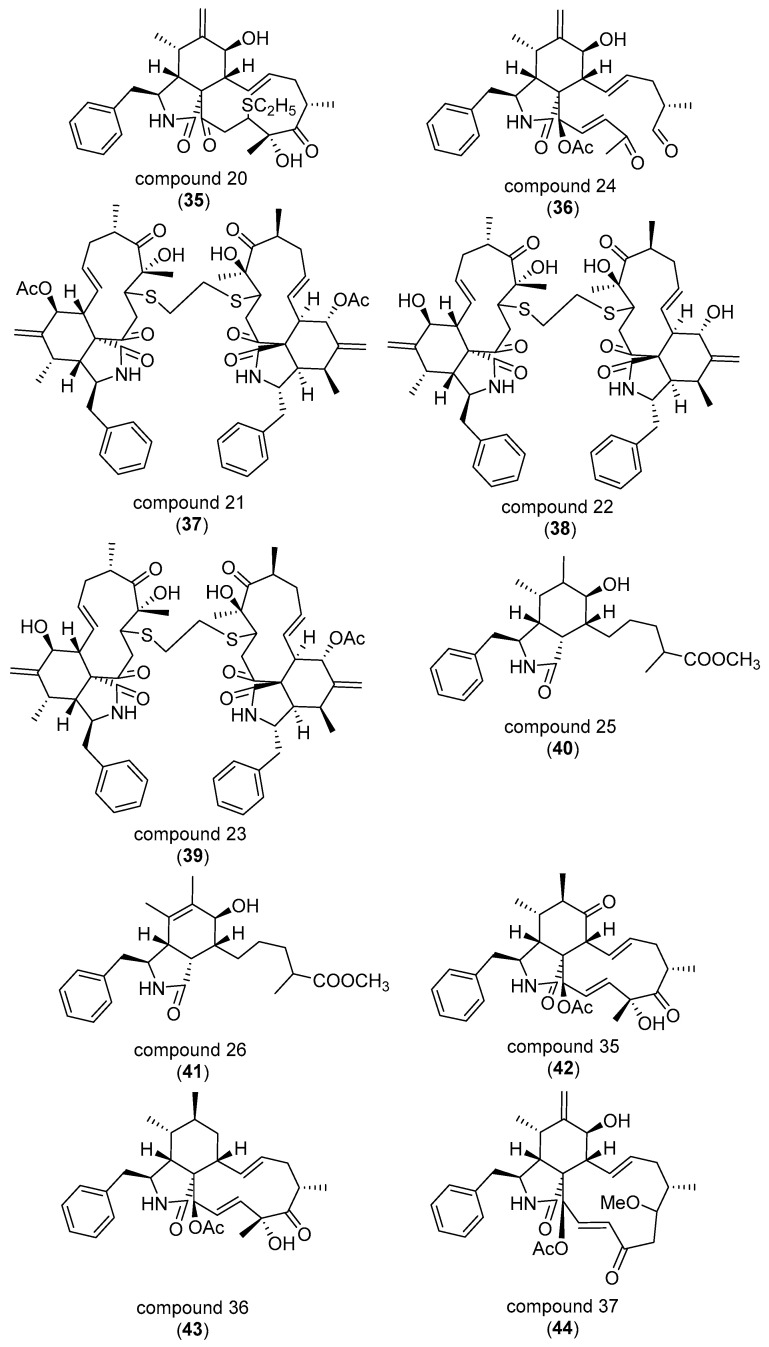
Structures of cytochalasin D derivatives (**9**–**44**) tested by Minato et al. (1978) [56].

The first attempts to quantify the effects exerted on cell cultures were made by Minato and coworkers (1973). Here, they tested a larger panel of cytochalasins (or zygosporins, see Figure 3) isolated from *Zygosporium masonii* and put the respective growth inhibition in HeLa cells into perspective with each other [56]. CD (**2**, = zygosporin A) and zygosporin D-G (**9**–**12**) were isolated and further semi-synthetically derivatized, totaling 37 examined cytochalasans (**2**, **9**–**44**), mainly differing in their oxygenation patterns. This revealed that the presence of an acetyl group at C-7 (considered as C-6 in [56], see Figure 3) as in zygosporin E acetate (**13**) correlated with a drastically reduced growth inhibition in comparison to a hydroxyl group at the same position as in zygosporin E (**10**) (effective dose, ED_50_ > 10 µg/mL vs. 0.4 µg/mL) [56]. This observation was later confirmed in a comparison of ChE (**7**) and ChE diacetate (**63**), although a lack of quantitative data makes the interpretation difficult [57]. Furthermore, an epoxy group between C-6 and C-7, as in cytochalasin F (CF, **48**), showed a higher growth inhibitory effect than CB (**1**) [58], indicating that the epoxy function is associated with an even more potent effect than the hydroxyl group [57,58,59]. The hydrogenation of the benzene ring (hexahydro cytochalasin D (**17**); ED_50_ = 0.23 µg/mL) to a cyclohexane ring (dodecahydro cytochalasin D (**16**); EC_50_ = 3.4 µg/mL) at C-3, in contrast, decreased cytotoxicity. The comparison of isocytochalasin D (**14**, hydroxyl group at C-21, ED_50_ > 10 µg/mL) with the corresponding 15-oxo compound (**15**, keto group at C-21, ED_50_ = 1.3 µg/mL) revealed a drastic increase in activity [56]. Cytochalasins with an opened macrocycle, on the other hand, were shown to exhibit reduced cytotoxicity (ED_50_ > 10 µg/mL; compound 25 (**40**) in [56]). This finding led Beno et al. (1977) to hypothesize that the macrocycle could serve as a “shield” within the binding site, with the inflexible isoindolone core mediating binding affinities to the target [60]. The importance of the macrocyclic ring in cytochalasins to confer cytotoxicity was later confirmed by [61], and especially a keto group at C-20 coincided with an increase in activity [58,59].

**Figure 4:**
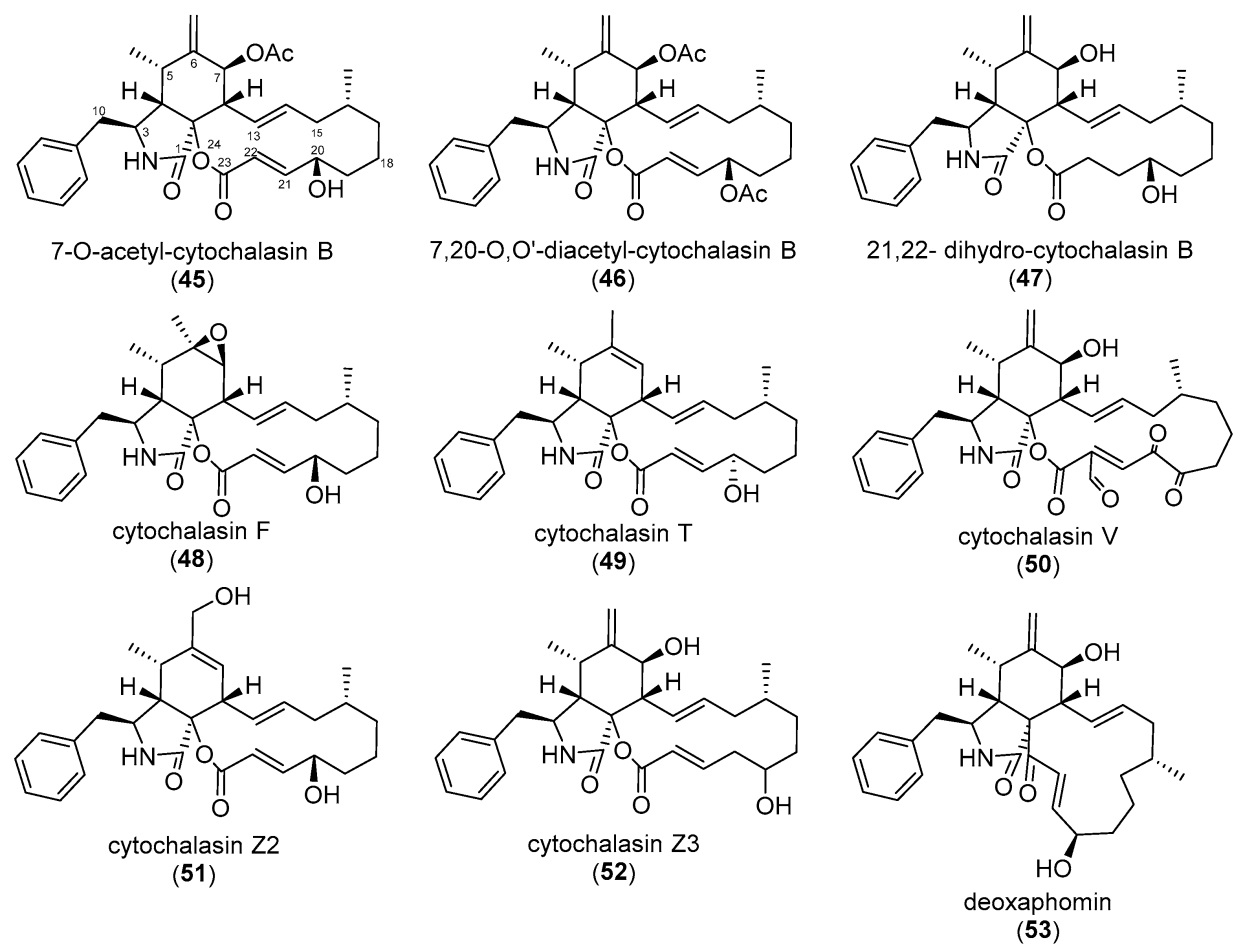
Structures of cytochalasans (**45**–**53**) investigated by van Goietsenoven et al. (2011) [58].

Another panel of eight cytochalasins of biological and semi-synthetic origin (CA (**3**), CB (**1**), 7-O-acetyl CB (**45**), 7,20-O,O-diacetyl CB (**46**), 21,22 dihydro CB (**47**), cytochalasin T (**49**), cytochalasin Z_2_ (CZ_2_, **51**), cytochalasin Z_3_ (**52**), and deoxaphomin (**53**); see Figure 2 and Figure 4) on six cancer cell lines displaying various levels of resistance (human OE21 esophageal, U373 glioblastoma, SKMEL28 melanoma, and A549 non-small cell lung cancer cell lines) or sensitivity (mouse B16F10 melanoma and human Hs683 oligodendroglioma cell lines) to proapoptotic stimuli, was explored later [58]. Here, an MTT-based colorimetric assay was employed for assessing cell metabolic activity to determine growth inhibitory concentrations (IC_50_) in vitro [62]. In this case, the authors concluded that 7-O-acetyl CB (**45**) exhibits a general, higher cytotoxicity than CB (**1**), which contrasts the observations by Minato et al. (1973) mentioned earlier, who postulated an increase in bioactivity upon hydroxylation of the C-7 position [56]. In any case, dissimilar, effective IC_50_ values were documented for each tested compound and experimental cell system, indicating different susceptibility profiles of the cell lines in use (compare, e.g., an IC_50_ = 3.5 µM for 7-O-acetyl CB (**45**) in B16F10 cells with an IC_50_ = 83 µM in SKMEL28 cells). Moreover, it could be shown that a chemical modification positioned at the perhydro-isoindolone core severely impacted cytotoxicity in CB (**1**, IC_50_ = 25.9 µM), CF (**48**, IC_50_ = 8.8 µM), and CZ_2_ (**51**, IC_50_ > 79.7 µM). The authors further speculated about a hydrolysis of the C-6/C-7 epoxy group, as found in CF (**48**), resulting in a diol system relevant to the bioactivity within the living cell [58]. However, no further data were presented to follow up on this hypothesis.

## 3. Correlating Cytochalasan Chemodiversity with the Activity Spectrum towards the Actin Cytoskeleton and Cell Morphology

To give context for the next set of experiments, the polymerization of globular actin (G-actin) to filamentous actin (F-actin) can be rationalized into three phases. First, a nucleation phase, where G-actin forms unstable trimers, which eventually elongate upon the addition of further G-actin subunits, creating thermodynamically more stable mini-filaments. All these processes occur in a concentration-dependent fashion. If the concentration of actin monomers exceeds a certain threshold (known as the critical concentration C_C_), a second phase follows, in which net actin assembly occurs (nucleation followed by elongation). This process is sustained as long as the concentration of remaining monomeric actin is sufficiently high. This gradually leads to a third, stationary phase, where further association and dissociation of monomers at both ends of the filament are in equilibrium with each other (treadmilling phase) [63]. In cells, therefore, individual filaments, or at least networks and bundles, display continuous turnover, although turnover rates in different subcellular actin structures are highly variable [64,65]. Importantly, G-actin has an intrinsic polarity that is translated into the filament, leading consequently to two filament ends with differential growth features. Each filament in cells or in vitro harbors a fast-growing, so-called barbed end and a slowly growing, pointed (or minus) end. The latter can be associated with a filament branch sprouting from the side of a mother filament associated with the Arp2/3 complex, a heteroheptameric complex harboring, aside from accessory factors, two **a**ctin-**r**elated **p**roteins (Arp2 and -3) [66]. The pointed end of a daughter filament can be separated from the mother filament by debranching factors. If free, the pointed end can also grow, but the C_C_ for elongation is higher at the pointed end than at the barbed end, meaning that at fixed actin monomer concentrations, growth is faster at the barbed end than at the pointed end.

**Figure 5:**
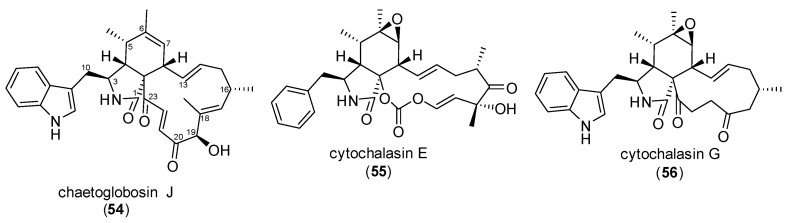
Structures of cytochalasans (**54**–**56**) tested by Löw et al. (1979) [67].

In addition to the discussed literature above reporting on *in cellulo* SARs, other authors like Löw and coworkers (1979) investigated CB (**1**), CD (**2**), cytochalasin E (CE, **55**), G (CG, **56**), ChA-C (**4**–**6**), ChE (**7**), ChF (**8**), and chaetoglobosin J (ChJ, **54**) for their ability to inhibit the polymerization of G to F-actin by viscometry (see Figure 2 and Figure 5) [67]. Weak effects were noted for CB (**1**), ChC (**6**), ChE (**7**), and ChF (**8**) at substoichiometric (0.4 mol/mol actin) concentrations, while ChA (**4**), ChB (**5**), and ChJ (**54**) effectively inhibited the increase in viscosity upon polymerization of actin. The reported curves showed a drastic reduction in total actin polymerization, reflected either by an earlier onset of the exponential growth phase (i.e., reduction in the critical concentration) or by reaching the stationary phase at earlier time points [67]. If focusing on SAR, the inhibition of actin polymerization was observed to a comparable extent for ChA (**4**) and CE (**55**), both of which share a double bond and a keto-group located in the macrocyclic ring [67], as well as the previously discussed epoxy group at positions C-6 and C-7 [58,59].

An exemplary electron micrograph of the highly potent ChJ (**54**) co-incubated with polymerized actin showed a drastic shortening of filaments, interpreted by the authors as filament degradation [67]. However, the mode of action of such shortening of filaments was later rationalized by MacLean-Fletcher and Pollard (1980) for CB (**1**) to be caused by a barbed end monomer addition interference mechanism [49], proposed to be conserved today among all cytochalasans.

**Figure 6:**
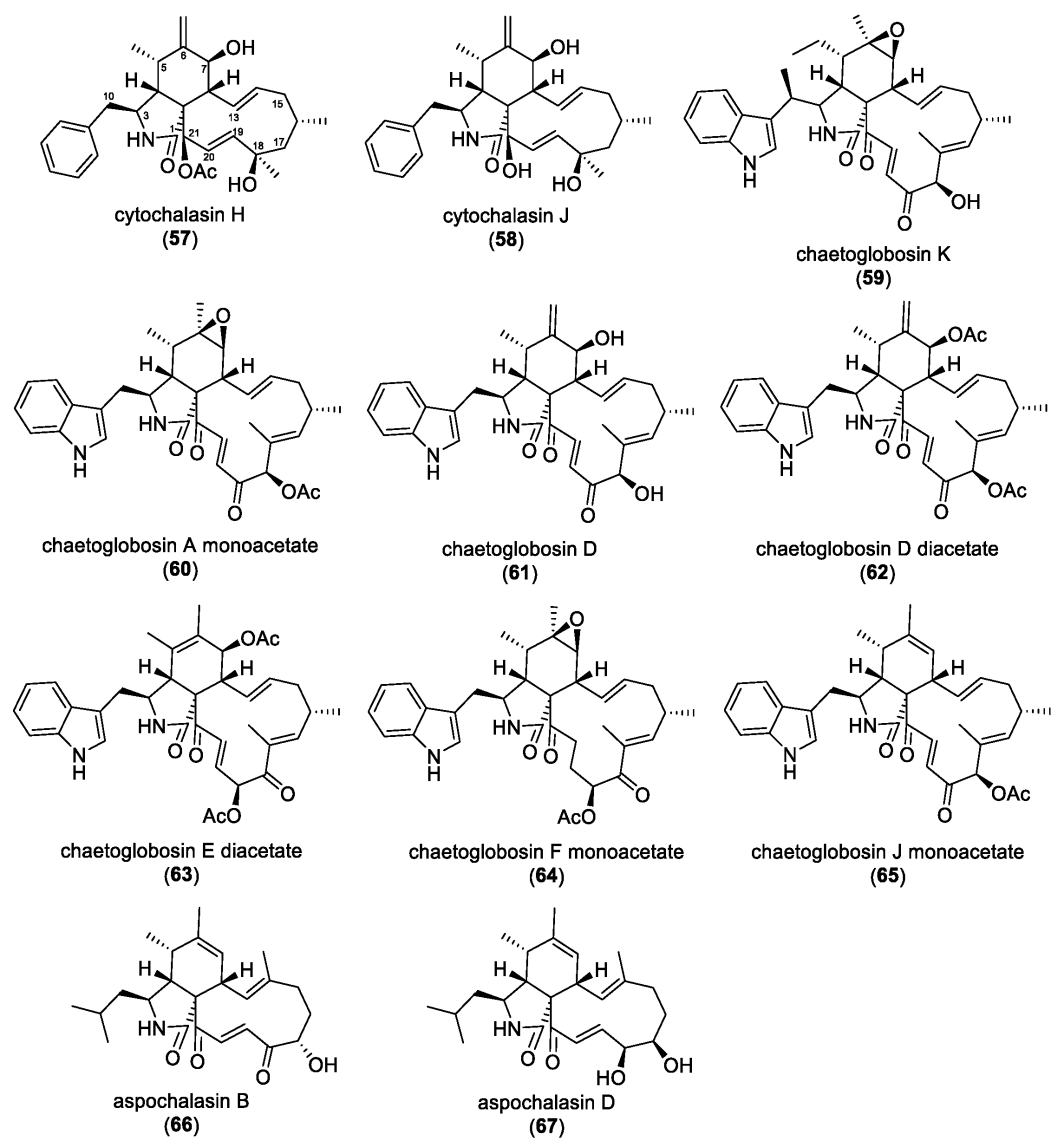
Structures of cytochalasans (**57**–**67**) tested by Yahara et al. (1982) [69]. The following compound names were interchangeably used by Sekita et al. (1985): 19-O-acetyl-chaetoglobosin A (**60**); 7,19-O-diacetyl-chaetoglobosin D (**62**); 7,19-O-diacetyl-chaetoglobosin E (**63**); 19-O-acetyl-chaetoglobosin F (**64**); 19-O-acetyl-chaetoglobosin J (**65**) [57].

In general, studies using CB (**1**) on tissue culture cells reported two types of concentration-dependent effects: (i) Inhibition of lamellipodia and membrane ruffles (actin-rich, flat protrusions or three-dimensional protuberances at the cell periphery), accompanied by abrogation of cell migration or size reduction of the lamella, the flat region proximal to the lamellipodium [68] after low-dose treatment (2 µg/mL) [69,70], and (ii) development of arborized and stellate cell morphologies after high-dose treatment (5–10 µg/mL) [69,70,71]. Visualization of the actin network using immunolabeling with actin-specific antibodies further showed that actin cytoskeletal components, such as stress fibers—anti-parallel bundles of myosin-bound actin filaments—largely disappeared. Furthermore, stellate cell shapes coinciding with star-like patches of actin aggregates were reported [47]. Yahara and coworkers used a similar actin visualization strategy for a larger body of cytochalasans (**1**–**8**, **23**, **53**–**67**, see Figure 2, Figure 3, Figure 5 and Figure 6) and complemented their efforts with viscosity assays [69]. Here, they could demonstrate a good correlation between the apparent actin binding affinities and growth inhibitory properties reported for CD (**2**, highly active) and, e.g., ChE diacetate (**63**) (less active). Additionally, the efficacy of tested cytochalasans concerning the induction of changes in cell morphology was documented by phase contrast and fluorescence microscopy, revealing that the inhibition of membrane ruffling is among the first effects (low dose effect), with the formation of “hairy” or “arborized” structures upon concentration increase (high dose effect), which is in accordance with the previously discussed data. High-dose effects were accompanied by the outer cell periphery collapsing towards the center of the cell, which had been interpreted as actin-dependent contraction. However, it should be noted that similar changes in cell and actin cytoskeleton morphologies can also be observed upon inhibition of myosin II-based contraction and its upstream regulator Rho [72,73], so we feel it is more likely that the retraction has occurred passively due to the loss of actin polymerization in adhesion-sensitive structures, which require myosin-based force to be maintained rather than retracted and disassembled. So, this phenotype would be analogous to the “retraction” induced by local inhibition of myosin II-based contractility in cells lacking microtubules (see, e.g., [73]). In other words, acute interference by these cytochalasans with actin assembly and thus the exertion of actin-based contractility through focal adhesions will have likely caused the cell collapse phenotypes observed [74]. However, this does not mean, of course, that high-dose cytochalasan effects are restricted to the inhibition of RhoA signaling and myosin II-based contractility. This notion is consistent with the fact that contractility inhibition by Y27632 (a Rho-kinase inhibitor), blebbistatin (myosin II inhibitor), or ML7 (myosin light-chain kinase inhibitor) each displayed effects additive to CB (**1**) [75]. In any case, the results indicated that an increased drug concentration causes morphological changes ranging from inhibition of membrane ruffling to the induction of cell rounding, with the only exception of chaetoglobosin K (ChK, **59**) being ineffective on the latter. The authors interpreted all these results to derive from the varying sensitivity of the common target protein to distinct cytochalasans [69]. Additionally, following treatment with ChA (**4**), ChB (**5**), chaetoglobosin D (ChD, **61**), ChJ (**54**), ChA monoacetate (**60**), and ChD diacetate (**62**), it was observed that cells are not able to spread again during a washout on the substratum after rounding, which was then shown to constitute a concentration-dependent effect. We encountered a similar phenomenon and attempted to estimate treatment conditions inferred from the extent of F-actin-disrupting effects [76,77,78,79]. Doing this, we observed that indeed there is a subset of compounds that tend to induce longer-lasting effects on F-actin networks, whereas other cytochalasans are again readily reversible. The physicochemical reasons for this behavior are unclear, but they seem to be linked to the modification of the macrocycle, which will be elaborated upon in a later passage [76,77,78,79].

**Figure 7:**
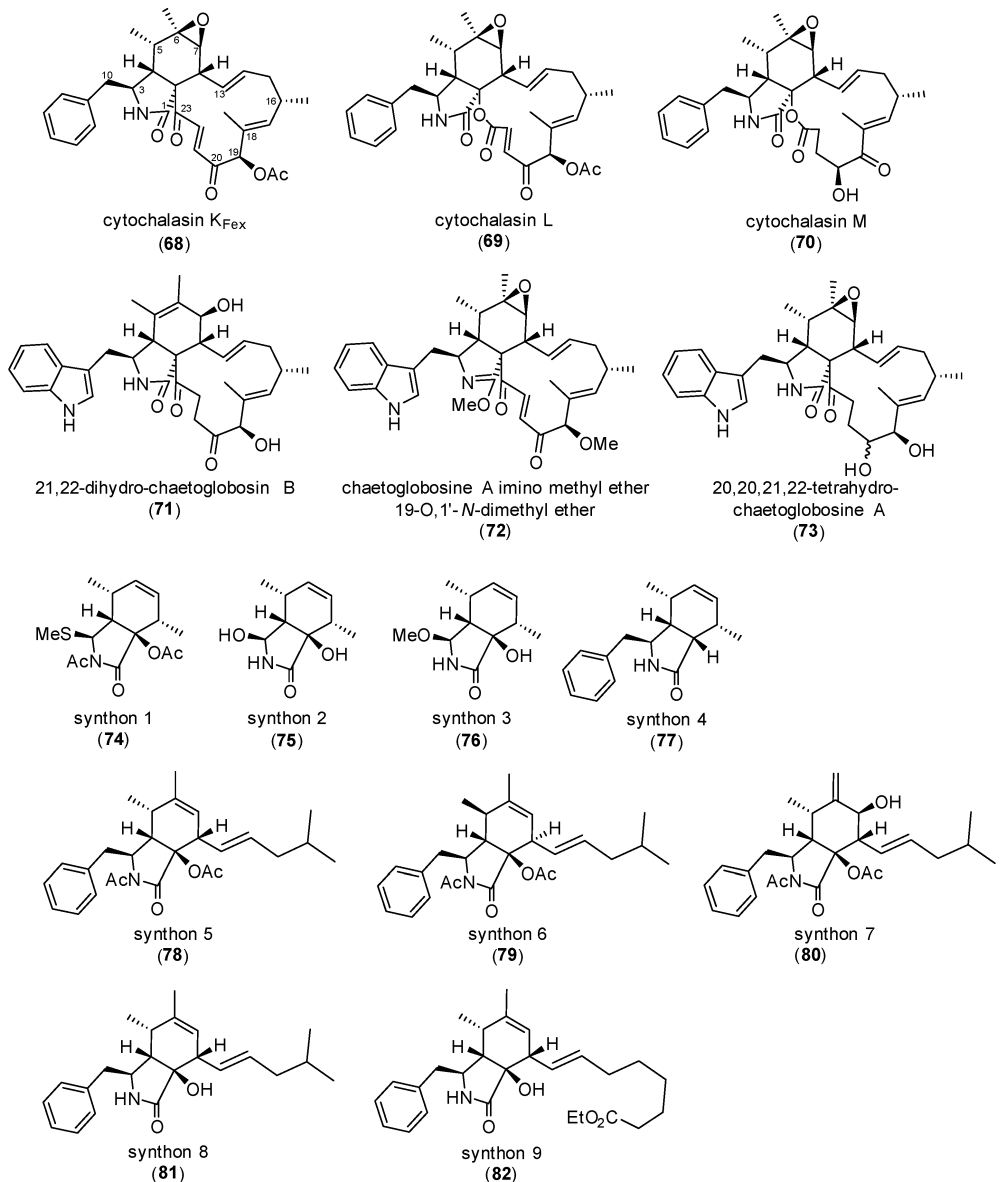
Structures of cytochalasans (**68**–**73**) and cytochalasin B synthones (**74**–**82**) analyzed by Sekita et al. (1985) [57].

In addition to the work of Yahara et al. (1982), additional SAR analyses were performed by Sekita and coworkers in 1985. The panel of previously tested cytochalasans was extended by three new cytochalasins (**68**–**70**), three derivatives of ChA (**72**, **73**) and ChB (**71**), and nine synthones of CB (**1**, **74**–**82**) (see Figure 2, Figure 3, Figure 5, Figure 6 and Figure 7) [57]. As noted before, a C-7 hydroxyl group [56,57,58] or an epoxy group at C-6 and C-7 [58,59] were indicative of potent bioactivity; however, compounds lacking oxygen decorations (e.g., ChJ (**54**), aspochalasin D (**67**)) in the perhydro-isoindolone core lead to a collapse of cellular integrity as well [57]. An important finding from this campaign was that the closed ring shape of the macrocyclic ring is a decisive feature. This conclusion was drawn after testing truncated CB (**1**) derivatives (synthones **74**–**82**; similar to [56,80]), which showed neither in vivo nor in vitro effects and were thus rendered inactive. The discussed structural deviations were deduced to act as contributors towards the interaction with a hypothesized hydrophobic binding site [57], as had also been speculated by others [56,67,81]. In addition, no strict correlation of bioactivity with specific functional groups at C-5 to C-7 (compare cytochalasin C (CC, **23**) and CD (**2**)) was observed. No impact on the bioactivity was also noticed when the macrocyclic ring was enlarged by oxygen insertion (from 13- to 14-membered; see cytochalasins K (**68**) and L (**69**)). Additional methylations at either C-10 or C-11 (see ChK (**59**)) revealed no significant implications for the bioactivity, either [57]. A direct comparison of cytochalasin K Fex (CK_Fex_ (**68**), named cytochalasin K in [57]) with its indoyl derivative 19-O acetyl ChA (**60**, named ChA monoacetate in [69]) showed that, aside from the higher effective concentration necessary to elicit observable effects for CK_Fex_ (**68**), each compound virtually led to the same phenotype in tissue culture cells. Moreover, it could be shown that lymphocyte capping is disturbed—a process dependent on heavy meromyosin (HMM), the larger fragment of myosin II motor proteins that is capable of binding actin, and together with actin contributing to a redistribution of antigen patches upon stimulus to form a “cap” on lymphocytes, detectable by using fluorescently-labeled antibodies [82]. Notably, Sekita and coworkers did not complement their data with immunofluorescence stainings, as was conducted in their previous papers [57,69]. It is possible that the authors have missed subtle differences between these compounds in the “low dose” concentration range exerted on the F-actin network, as solely “overdosed” samples were employed for addressing the cytochalasans’ bioactivity. In summary, we conclude that the amino acid substituent at the C-10 position apparently affected the general bioactivity without changing the MoA, thus acting as a modulator of cytochalasans’ affinity for actin.

**Figure 8:**
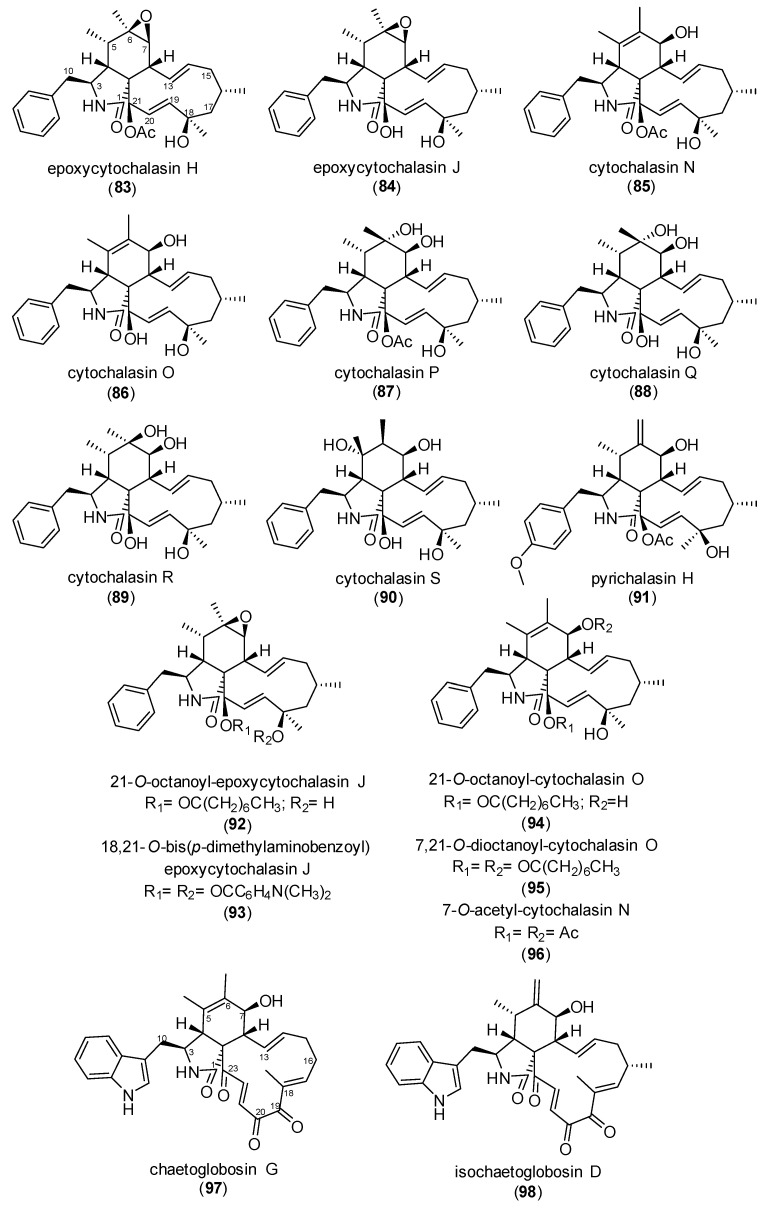
Structures of cytochalasans (**83**–**96**) tested by Hirose et al. (1985) [85] and chaetoglobosin G (**97**) and isochaetoglobosin D (**98**) by Thohinung et al. 2010 [92].

The influence of the hydroxyl group described at C-7 associated with compounds sharing a higher efficacy [56,57,58] and corresponding lipophilicity impacting effectiveness, as, e.g., previously hypothesized by [57], was further addressed by Hirose et al. (1990). In this study, the authors tested 19 cytochalasans (**2**, **4**, **57**, **58**, **83**–**96**), six of which were new (**85**–**90**), mainly deviating in the oxygenation pattern of their perhydro-isoindolone core, using previously described methods (Figure 2, Figure 6 and Figure 8) [57,69]. However, for the first time, a fluorescence-based assay (pyrene assay, [83,84]) was used to examine cytochalasan bioactivity on actin assembly instead of viscometry [85], directly documenting inhibitory effects on actin polymerization. Compounds including a 6,7-epoxide (epoxycytochalasin H (**83**) and J (**84**)), a 6(12)-en-7-ol (cytochalasin H (**57**) and J (**58**), or a 5-en-7-ol moiety (cytochalasin N (**85**) and O (**86**)) all exhibited effects to comparable extents. This showed that neither hydroxylation, acetylation, nor the presence of a keto-group at C-21 was associated with a significant net impact on the extent of the respective cytochalasin’s bioactivity harboring this specific backbone. The bioactivity of the novel 5,7- or 6,7-diol cytochalasins P-S (**87**–**90**) was relatively weak or even absent. The authors assumed that the conformational change of the perhydro-isoindolone core, induced by the introduction of an additional hydroxyl group, was detrimental to its interaction with the binding site. Moreover, pyrichalasin H (**91**), a cytochalasan with a *p*-methoxyphenyl group at C-3 [86], showed an activity similar to cytochalasin H (**57**, phenyl at C-3), suggesting that the methoxy group does not alter the activity in this particular cytochalasan backbone. Furthermore, derivatives synthesized with different hydrophobic groups introduced to C-7/C-21 and C-18/C-21 (see compounds 11–15 (**92**–**96**)) did not enhance their bioactivities, indicating that an artificial increase or decrease in lipophilicity had little effect, at least in these experimental conditions [85]. Further work on characterizing the interference of CD (**2**) with actin dynamics, ChA-F (**4**–**8**, **61**), and ChJ (**54**) (Figure 2, Figure 5 and Figure 6) was accomplished by Maruyama and coworkers utilizing pyrene assays [87]. The authors confirmed previous findings based on viscometry [67], revealing that ChA (**4**), ChB (**5**), ChJ (**54**), and also ChD (**61**) strongly decreased actin polymerization to almost zero. ChC (**6**), ChE (**7**), and ChF (**8**), however, displayed hardly any effect. Interestingly, higher concentrations of CD (**2**) reduced the lag phase commonly seen with actin polymerization, which has been interpreted as a nucleation-enhancing effect [88]. This phenomenon was already noticed by Brenner and Korn (1979) and explains, in retrospect, the curve shifts caused by ChE (**7**) and ChF (**8**) as well as CB (**1**) reported by Löw et al. [67,89]. Further studies examining the effect of ChJ (**54**) on HMM-decorated actin filaments showed that this compound reduced elongation at the barbed end [87]. At the same time, the G- to F-actin equilibrium was shifted towards the pool of G-actin in the presence of stoichiometric concentrations of ChJ (**54**), as shown by the sedimentation patterns of G- and F-actin after ultracentrifugation and, amongst others, an assay assessing G-actin binding to DNase I [87,90,91]. Furthermore, the authors reported on a compromised actin–myosin interaction in the presence of ChJ (**54**) if pre-treated actin is mixed with myosin, which was explained by a conformational change of F-actin, leading to impaired myosin binding [87]. It would be interesting to study if this conformational change imposed on actin filaments is persistent over time, as this could serve as a puzzle piece explaining the irreversible effects of ChJ (**54**) in certain concentration ranges reported by Yahara et al. [69] and others [78] or, e.g., in the case of cytochalasin S (CS, **90**) effecting lymphocyte capping activity in the absence of F-actin polymerization inhibition [85].

At this point, it is important to note that only a subset of the previously discussed studies [57,69,85] chose analogous methods and cytochalasans, rendering them well comparable. Observations concerning morphological changes, for example, were generally in good accord. However, one issue to point out is that Yahara and coworkers (1982) used maximal effects with concentrations tested at 0.2 to 20 µM and incubation times ranging from 0.5 h up to 2 h for their qualitative descriptions. In contrast, Hirose et al. and Sekita et al. [57,85] did not explicitly mention the tested concentrations but chose shorter incubation times (from 0.5–1 h [57] and 0.5 h [85]). Peculiarly, CS (**90**) showed no activity in the in vitro actin assays, despite being reported to form hairy actin-containing structures in cells and to inhibit lymphocyte capping [85]. If it were correct that cytochalasin exhibits no activity on actin on its own, the inhibitory effect on lymphocyte capping could possibly be explained by interference with the interplay between actin and HMM (see above). Such potential secondary effects will be further discussed in a later section of this review. To further expand on the comparability of employed methods, we note that the reported inhibition rate of, e.g., CD (**2**) strongly depended on the method chosen (72% decrease in total extent of F-actin polymerization measured with viscometry [57,69], vs. 36% measured with pyrene assay [85]). Differences were also seen depending on whether polymerization was induced with KCl, MgCl_2_, or both [49]. Cell line-dependent differences in susceptibility to distinct cytochalasans (see [58]) were already noted by Thohinung and coworkers (2010), who compared the effects of ChC (**6**), ChD (**61**), ChF (**8**), chaetoglobosin G (**97**), and isochaetoglobosin D (**98**) (Figure 2, Figure 6 and Figure 8) on KKU-100 and KKU-OCA17 cells [92]. As elaborated on above, we have encountered additional examples when comparing quantitative data on growth inhibitory concentrations using distinct cell culture models.

In summary, the majority of published literature on the effects of different cytochalasans still suffers from a lack of unified test strategies, concentrations, or cellular model systems (see [55,56,57,59,61,67,69,85,93,94,95] to cite a few), although this is about to change in the near future (see below). Nevertheless, the aforementioned studies proved to be highly valuable for qualitative comparisons. From the knowledge gathered throughout the past decades, it can be concluded that the oxidation status of closely related cytochalasans affects the strength of their bioactivity [57,58,59], which further extends to an intact macrocyclic ring [56,57,60] and the presence of an aromatic system attached to the pyrrole moiety [56].

**Figure 9:**
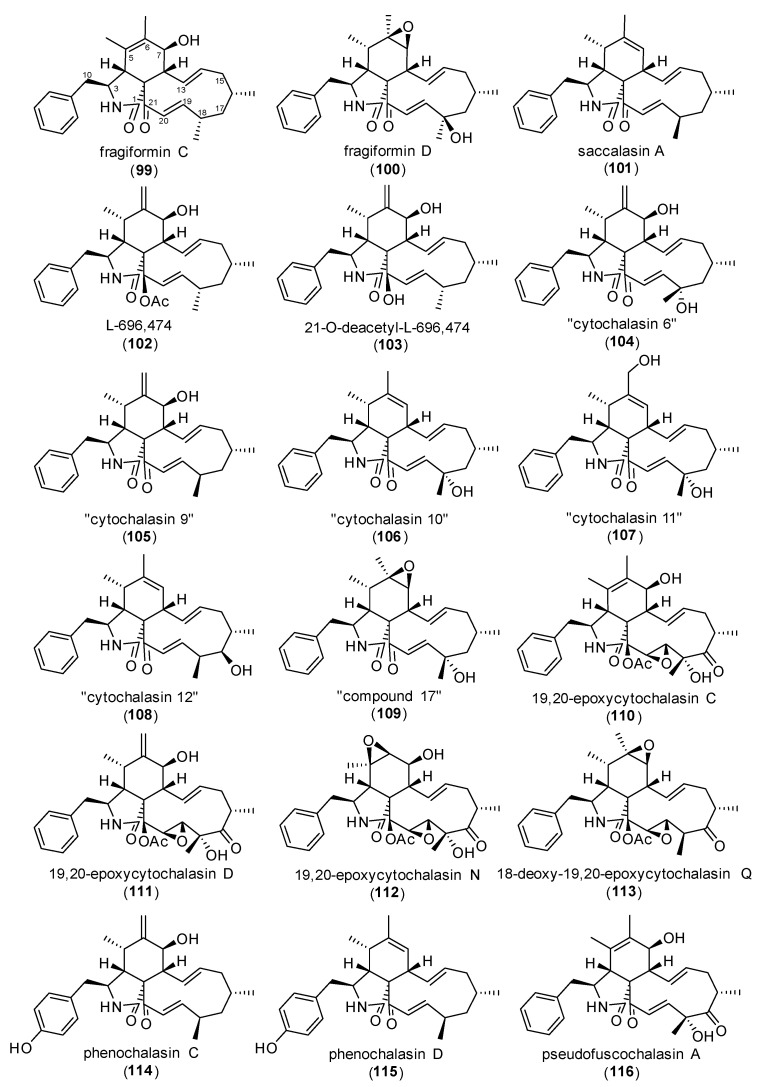
Structures of cytochalasans tested by Kretz et al. (2010) (**99**–**115**) and Lambert et al. (2021) (**116**) [76,78].

Our recently published natural product isolation campaigns using a strain of the Dothideomycete *Sparticola triseptata* and other Sordariomycetes strived to improve the comparability of treatment conditions. A large panel of cytochalasans (**1**, **2**, **4**, **48**, **51**, **53**, **57**, **61**, **99**–**115;** see Figure 2, Figure 4, Figure 6 and Figure 9) reported by [78] was tested for its actin network disruptive capabilities in human osteosarcoma cells (U-2 OS), which was monitored by visualization of the actin cytoskeleton with fluorescently-labeled phalloidin. The results suggested that an α-β unsaturated bond between C-19 and C-20 in the macrocycle next to the ketone affected reversibility [78]. This was later corroborated by [76] reporting on pseudofuscochalasin A (**116**), a deacetylated and reduced derivative of CC (**23**).

**Figure 10:**
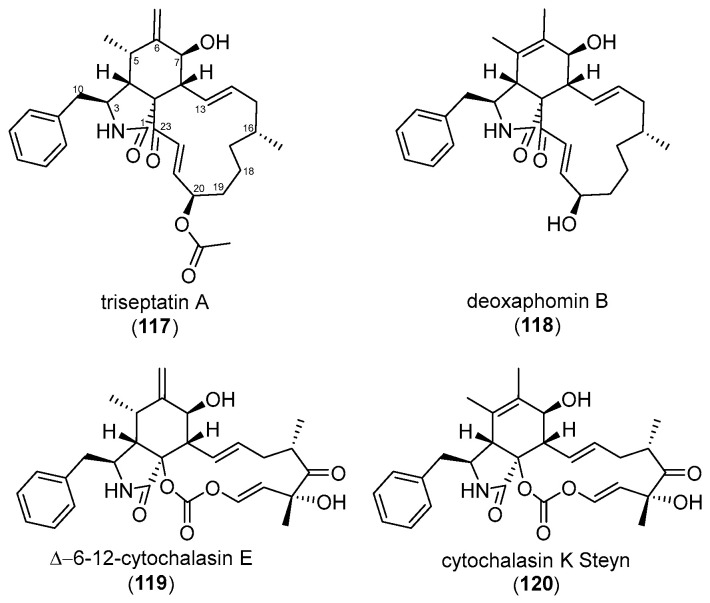
Structures of cytochalasans investigated by Garcia et al. (2022) (**117**, **118**) and Pourmoghaddam et al. (2022) (**119**, **120**) [77,79].

Recently, deoxaphomin B (**118**) (see Figure 10) was re-isolated by [77] and compared with the F-actin-network-disrupting effect of deoxaphomin (**53**) reported by [78] in U-2 OS cells. This revealed that, as opposed to deoxaphomin (**53**), the disruption of the F-actin network caused by deoxaphomin B (**118**) is fully reversible [78]. However, both compounds share the α-β unsaturated bond between C-19 and C-20, previously shown to be a causative determinant for reversibility. Hence, multiple structural features can contribute to a cytochalasan’s ability to impose irreversible changes to the F-actin network, dependent on chemical context [77].

**Figure 11:**
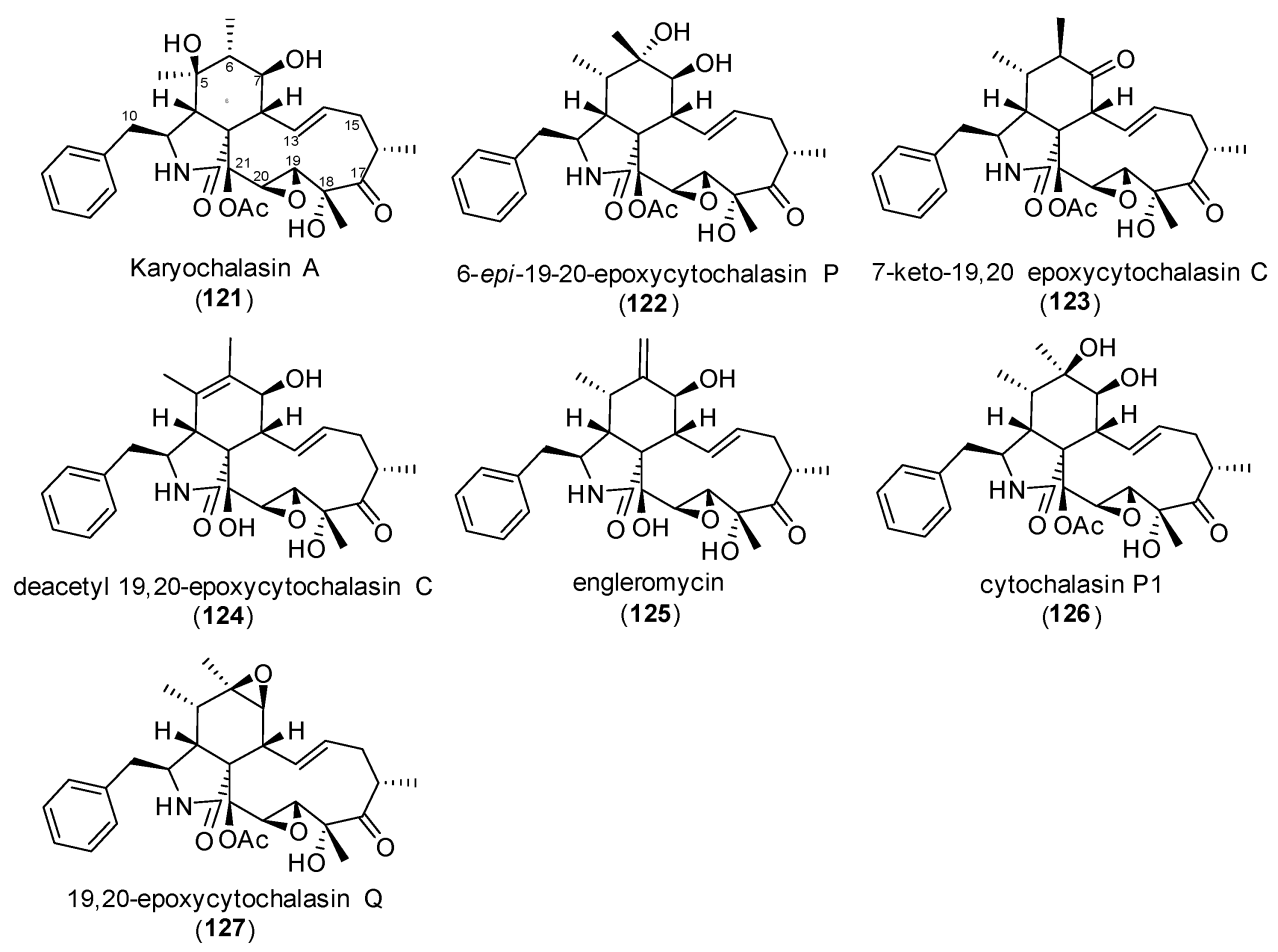
Structures of cytochalasans (**121**–**127**) tested by Lambert et al. (2023) [96].

Another recent study [79] reported that the methyl groups in a steric position planar to the six-ring in cytochalasin K_Steyn_ (**120**, named cytochalasin K in [79]; see Figure 10) were associated with a drastic decrease in activity. This pattern of decreased activity could not be confirmed for this specific configuration in a panel of recently reported cytochalasins sharing an epoxidated macrocyclic backbone (**110**–**112**, **121**–**127**, see Figure 9 and Figure 11) isolated from cultures of *Xylaria karyophthora* [96]. However, the steric conformation of the hydroxyl groups attached to the six rings was shown to be important. Thus, it seems necessary to regard the backbone of the analyzed cytochalasan core structures separately from its oxidative status for now, until more information on how ring size and steric configurations affect binding activity is available. In any case, studies focusing on structural aspects of the actin–cytochalasin interaction are desperately needed, with the hope of being able to better correlate previously published observations with each other and with respect to the bioactive moieties of respective cytochalasans. Curiously, a subset of cytochalasins in Figure 11 has been shown to be non-toxic towards L929 mouse fibroblast cells in the commonly tested concentration ranges, even though they are still active against other cancer cells and are even able to exert detrimental effects on the organization of the F-actin network in U-2OS cells [96]. It can only be speculated on what the mechanistic reason for this behavior could be. It has already been hypothesized that chemical modifications could hamper the cytochalasans’ ability to pass the plasma membrane [69]; however, conclusive data demonstrating this remain to be reported.

**Figure 12:**
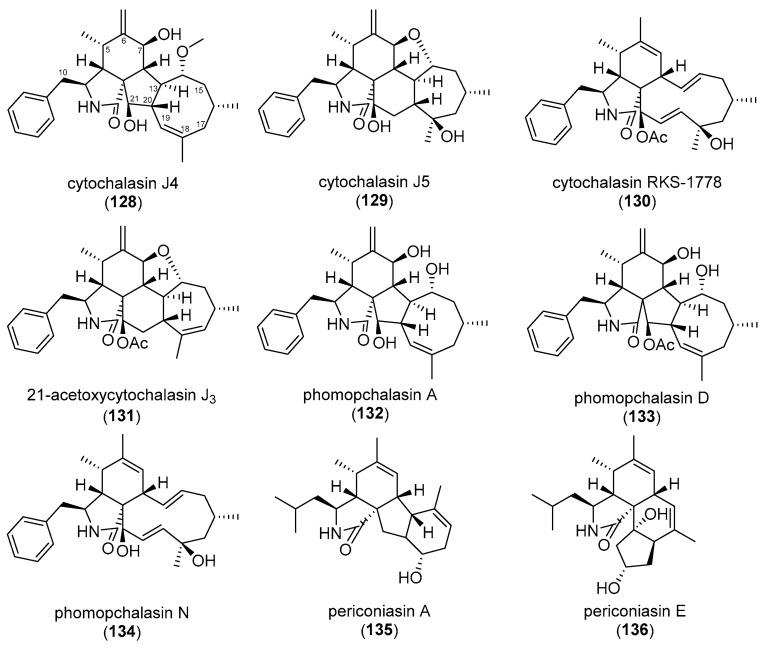
Structures of cytochalasins (**128**–**134**) analyzed by Kemkuignou et al. (2022) and periconiasin A and E (**135**–**136**) [97,99,100].

Recently, cytochalasins (**57**, **58**, **128**–**134**, see Figure 6 and Figure 12) isolated from *Diaporthe* spp. have been reported that were rendered inactive by conversion under acidic conditions [97]. It is unclear at present if this apparent inactivity is due to interference with its ability to bind to actin or its inability to enter the cell. The same formal proof is lacking for the formerly discussed, less active cytochalasins reported in [56,57,77,78,79,96].

**Scheme 1:**
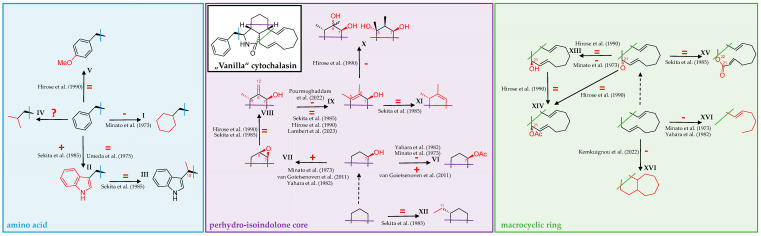
Over the past five decades, a multitude of cytochalasin derivatives has been shown to harbor differential efficacy when tested for cytotoxicity and bioactivity in functional cell biological assays towards actin-dependent structures and actin polymerization in biochemical experiments. Modifications at the incorporated amino acid (light-blue box, **left**), perhydro-isoindolone core (purple box, **middle**), and macrocyclic ring (light green box, **right**) have by and large been independently regarded from the remaining cytochalasan backbone (blue, purple, and green lines drawn into a “Vanilla” cytochalasin in the **middle top-left**). Derivatives featuring a de-aromatized phenylalanine unit were shown to elicit reduced activity (**I**). Tryptophane-derived indole-substituted compounds were reported to either increase or have no effect on bioactivity (**II**), while other non-ribosomal peptide substituents have so far not been comprehensively studied (**IV**). No effect on bioactivity was found for a methyl attached to C-10 (**III**), or a methoxy group substituted in *para* position at the phenyl shown in **V**. Acetylation of a C-7 hydroxy group found in the perhydro-isoindolone core was shown to either decrease or increase efficacy (**VI**), while substitution with a C-6/C-7 epoxide increased activity (**VII**). Toxicity was unchanged for a combination of C-7 hydroxy, C-6 methylene, and C-5 methyl moieties (**VIII**), while a double bond between C-5 and C-6 either had no influence or decreased efficacy compared to the former (**IX**). Epimers of further hydroxylated compounds were shown to display decreased activity (**X**). However, compounds lacking oxygen functions in the perhydro-isoindolone core did not differ in efficacy compared to oxygenated ones (**XI**). The same was reported for a methylated methyl attached to C-11 (**XII**). Hydroxylation of a keto-group at C-21 was without effect or decreased efficacy (**XIII**), while acetylation did not change activity, neither when compared to a ketone nor a hydroxy group at C-21 (**XIV**). Enlargement of the macrocyclic ring by oxygen insertion, forming a lactone, was also without effect (**XV**). Finally, ring opening and polycyclization were both reported to decrease activity (**XVI**). For conflicting reports and further structural details, see main text above. Studies cited in the scheme are as follows: Umeda et al. (1975) [55], Minato et al. (1973) [56], Sekita et al. (1985) [57], van Goietsenowen et al. (2011) [58], Yahara et al. (1982) [69], Pourmoghaddham et al. (2022) [78], Hirose et al. (1990) [85], Lambert et al. (2023) [96] and Kemkuignou et al. (2022) [97].

Before summarizing this section, we would like to give the reader the opportunity to reflect upon the most conspicuous findings elaborated on in chapters 2 and 3 above. In Scheme 1, we have sought to summarize how distinct structural modifications of a “Vanilla” cytochalasin, separated into three main hypothetical structural elements, impact bioactivity.

Summarizing this section, we conclude that a re-introduction of the previously reported bioactive cytochalasans and newly generated ones derived from a singular backbone by means of medicinal chemistry in standardized cytotoxicity assays, backed up by functional cell biological and biochemical experiments, seems to be the most promising way to obtain a comprehensive SAR. Recent findings reported by [76,77,78,79,97,98] largely corroborated the qualitative data acquired and discussed in previous decades, showing that there are indeed cell-type-specific effects that occur in standardized assays [58,96]. Furthermore, we conclude that the strength of bioactivity of a given cytochalasan seems to not exclusively rely on the presence of specific oxidation patterns or chemical moieties, but instead to be co-dependent on an intact macrocyclic ring [56,60,97] and to moderately depend on an aromatic system attached to the perhydro-isoindolone core [56]. This raises the question if cytochalasans incorporating other substituents than a phenyl, a *p*-methoxyphenyl, or tryptophane-derived indol moieties, such as in the aspochalasin (**66**, **67**) and the periconiasin series (exemplified by **135** and **136**, [99,100]), cause the same effects in functional assays using living cells. This question has so far, to the best of our knowledge, not yet been addressed. Depending on the size and composition of the macrocycle, even minor changes to the configuration of the 6-ring attached to the pyrrole seem to have drastic effects on bioactivity [79], while for other macrocyclic ring configurations, alternative modifications are necessary to cause measurable effects. Examples of this would be the previously discussed reduction in bioactivity for CK (**120**) or the alleviated cytotoxicity of epimers of the 19,20-epoxycytochalasin series isolated by Lambert et al. (2023) [96]. This notion extends to the apparent irreversible effects exerted by compounds such as deoxaphomin B, ChgB, CE, and pseudofuscochalasin A (**118**, **5**, **55**, **116**) that seem to equally depend on specific, but not yet fully understood, configurations of the cytochalasan backbone [76,77,78,79]. The pyrrole unit, an integral part of cytochalasans, seems to be of utmost importance, as naturally occurring modifications reported from fungal natural product screening campaigns are scarce. ChK (**59**) has been reported as an example of a cytochalasan bearing a modification at the carbon bridge connecting the indole to the pyrrole unit; however, data linking its ability to inhibit actin filament formation in vitro with its cellular cytotoxicity are still lacking [69]. Finally, the reason for changes in bioactivity, be it a reduction or increase in affinity for a given cellular target or a change in physicochemical characteristics affecting cell permeability, has yet to be addressed.

## 4. Cytochalasins Interfere with Cytoskeletal Dynamics

**Figure 13:**
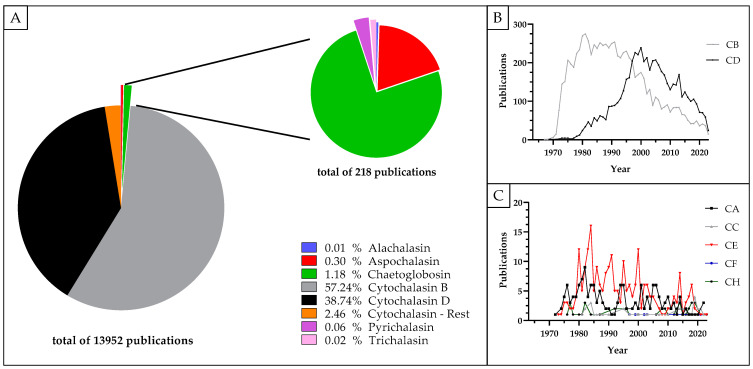
(**A**). Number of indexed publications in a PubMed literature search for different members of the cytochalasans. (**B**). Number of indexed publications in a PubMed literature search for CB (**1**) vs. CD (**2**) between January 1967 and June 2023. (**C**). Number of indexed publications in a PubMed literature search for CA (**1**), CC (**23**), CE (**55**), CF (**48**), and CH (**57**) between January 1967 and June 2023. To search for indexed publications for different cytochalasan subclasses, the keywords “alachalasin”, “aspochalasin”, “chaetoglobosin”, “pyrichalasin”, and “trichalasin” were used. For the remainder, “cytochalasin A” was used for CA (**1**), “cytochalasin B” for CB (**2**), “cytochalasin C” for CC (**23**), “cytochalasin F” for CF (**48**), and “cytochalasin H” for CH (**57**), spanning a time interval from 1967 until June 2023. Note the overwhelming share of publications indexing either CB (**1**) or CD (**2**).

Independent of the previously mentioned wealth of cytochalasans to select from to interfere with F-actin and thus cytoskeletal dynamics for the purpose of using them as tool compounds, most studies currently are still focusing on a much smaller subset of cytochalasans, with CB (**1**) and CD (**2**) being by far the most widely used ones. To ask how relevant cytochalasans are in modern times, we conducted a database search in PubMed, summarized in Figure 13. Retrieved PubMed entries demonstrated a specific focus on CB (**1**) and CD (**2**), while the third-most investigated cytochalasin, CE (**55**), still received only a small fraction of the attention from the scientific community as compared to CB (**1**) and CD (**2**) (Figure 13A–C). However, all other subclasses of cytochalasans (alachalasins, aspochalasins, chateoglobosins, pyrichalasins, and trichalasins) were again even less frequently studied or employed (Figure 13A). The depicted statistics clearly reveal how predominantly CB (**1**) and CD (**2**) have been used in comparison to other cytochalasins in the past (compare Figure 13B,C), with a steady decline in publications keywording these two compounds in more recent years (Figure 13B). While combing through the literature, we noticed that many papers that we used for the preparation of this review were not indexed by PubMed and could instead be found in a “Google Scholar” search. For example, highly cited articles from Bear et al. (2002, Cell) [101], Pruyne et al. (2002, Science) [102], and Mehidi et al. (2021, Nature Cell Biology) [103] could not be found when using the keywords “cytochalasin” and “VASP” for Bear et al. (2002); “cytochalasin” and “spectrin” for Pruyne et al. (2002); “cytochalasin” and “WRC” for Mehidi et al. (2021). Scientists wishing to read on the topic should keep this in mind when doing their literature research. This means that the numbers displayed in Figure 13 are likely underestimations.

Cytochalasans are commonly applied as tool compounds in the context of research, raising questions about how intact actin architectural organization and its dynamics contribute to different cellular processes. In most cases, they are employed to interfere with specific actin-dependent structures or activities and, in some cases, can even increase specific features, such as fenestrae numbers in liver sinusoidal endothelial cells (LSEC) [104]. The eukaryotic actin cytoskeleton is well characterized as the driving engine of many force-dependent processes, both initiating and maintaining all sorts of movements of cells and within them, such as endocytosis, cytokinesis, and migration of cells or whole tissues [105]. In order to exert physical forces on the plasma membrane, e.g., to push it forward, structures such as lamellipodia—sheet-like protrusions located at the periphery of the leading edge—are built. Pulling forces, instead, drag the cell body along, during directional migration [106]. In pushing or pulling actin filament networks or bundles, such as lamellipodia/filopodia and focal adhesions, respectively, the plus ends of filaments are pointing towards the membrane, while the minus ends point in the opposite direction. Polymerization and depolymerization by actin monomer addition and removal at the barbed and pointed ends, respectively, are tightly regulated by ABPs, facilitating dynamic reconstruction and maintenance of all actin-dependent macrostructures in cells [107]. Aside from the protrusive lamellipodia and filopodia, contractile structures such as stress fibers are anchored in aforementioned focal adhesions. Filopodia constitute finger-like protrusions emerging from the plasma membrane and contain parallel bundles of actin filaments, whereas stress fibers largely comprise anti-parallel filaments that mediate contractility through a pseudo-sarcomeric arrangement [37]. A subtype of protrusive bundles that can be distinguished from filopodia are microspikes, which are finger-shaped like filopodia but always remain largely embedded into the lamellipodium network without protruding beyond the plasma membrane [108]. This distinction means that, per definition, microspikes cannot exist without lamellipodia [109,110], while filopodia are functionally fully separable from the former [111,112].

Monomeric, polymerizable actin is abundantly present in cells, with estimated concentrations of up to 300 µM [113]. Although this is thought to be required for building novel and/or the turnover of existing actin structures in cells, the spontaneous assembly of these monomers into filaments must be tightly controlled, if not entirely inhibited. This notion conceptually demands exquisite control over the pool of available, polymerizable actin, as well as its assembly into double-helical filaments or temporally and spatially controlled disassembly [114,115]. Inhibition of spontaneous polymerization is thought to be mediated through actin monomer-binding factors, such as profilins or β-thymosins, the most abundant of which in humans appears to be thymosin-β4, but the precise cell biological functions of all these factors are far from being fully understood.

Profilin is an actin monomer-binding protein that is thought to prevent unwanted nucleation and furthermore acts as a regulator of actin dynamics in combination with actin nucleating and elongating factors such as formins (e.g., mDIA1-3, FMNL2/3; [116]) or Ena/VASP family proteins [117]. Numerous studies performed with purified proteins in vitro have led to a number of assumptions that can be summarized as follows: Purified profilin and VASP, for example, strongly enhance filament elongation rates in a context-dependent fashion, i.e., independently of the ionic environment, while VASP alone requires a low ionic environment to cause comparable effects [118,119,120,121]. Additionally, VASP was described to prevent the capping of filament barbed ends by heterodimeric capping (CP) protein [101,105,118,119,120,122], but the precise regulation of this proposed antagonism in cells has remained incompletely understood. CP, on the other hand, is assumed to inhibit filament elongation but was at the same time proposed to increase the branching density in networks of actin filaments generated by the Arp2/3 complex. This activity was considered to keep filaments short and rigid rather than long and fragile [123,124]. A more recent study, though, sought to explain the promotion of Arp2/3 complex-dependent actin network formation by CP to derive from the ability of the latter to remove a non-productive interaction of WAVE-type NPFs (nucleation-promoting factors) with the barbed ends of actin filaments, mediated by the so-called β-tentacle. Truncation of this fragment on the β-subunit of CP not only abrogated Arp2/3-dependent actin assembly *in vitro*, but also in lamellipodia, i.e., the complex environment of the entire cell [125], suggesting CP activities to certainly be more complex than previously anticipated. On top of that, single filament total internal reflection fluorescence microscopy (TIRF-M) experiments revealed that CP can synergistically cooperate with cofilin, allowing the disassembly of actin filaments at both ends while funneling G-actin onto uncapped actin filaments, thereby increasing actin turnover [126,127]. On the other hand, capping protein was previously also described to inhibit bursts of actin filament depolymerization mediated by the three-component actin disassembly system comprising cofilin, coronin, and Aip1 [128]. To grasp the complexity of all these observations in detail, the interested reader is guided towards recent, excellent, comprehensive reviews summarizing the state of the art of our understanding of various aspects of this and other exciting components of the actin machinery, which have appeared over the last couple of years [37,38,105,129,130,131]. Together, the intricate mechanisms of maintenance of these complex machineries are only beginning to be elucidated in the complex environment of the cell cytoplasm. Considering that studies assessing how cytochalasans interfere with actin assembly mechanistically have historically been carried out by employing biochemical approaches, it has remained underexplored, aside from their direct effects on polymerization, how cytochalasan–actin interactions in cells interfere with the function and activities of the multitude of actin regulatory proteins.

The cytochalasan–actin interaction is commonly described to comprise a barbed end interference mechanism, leading to “capping” of the barbed end with high affinity, drastically slowing down (but perhaps not entirely stopping [11,132]) the elongation of actin filaments with high affinity (Kd: ~2 nM for CD, **2**; [54]). Other biochemical effects were noted at higher concentrations, such as 2–20 µM, where CD (**2**) was shown to bind and dimerize G-actin, thus acting as a nucleator [54,133]. Furthermore, the binding of CD (**2**) to ATP-actin was shown to lead to an increased hydrolysis of F-actin-bound ATP to ADP and orthophosphate, interpreted as accelerated filament aging, which may potentially affect F-actin–ABP interactions depending on the phosphorylation status of the nucleotide [11,54,133]. Of note, the F-actin severing and disassembly activities of ADF/cofilin family members were previously described as enhanced on ADP-actin filaments vs. ATP-F-actin [134]. Intriguingly, in this context, other studies indeed observed filament severing upon treatment with CD (**2**) [54,135,136,137,138]. In any case, the precise binding site of CD (**2**) on actin has so far only been described for G-actin, but not F-actin, via co-crystallization of G-actin with CD (**2**) and subsequent X-ray scattering, showing the ligand in a hydrophobic cleft between actin subdomains 1 and 3 [139]. This binding site of CD (**2**) with actin was described to overlap with the binding motifs of other ABPs, such as gelsolin [140], the *Drosophila* three β-thymosin repeat protein ciboulot [141], vitamin-D-binding protein [142,143], the formin homology 2 (FH2) domain of formins [144], the G-actin-binding (GAB) domain of VASP [117], and the Wiskott–Aldrich syndrome protein (WASP) homology (WH)-2 domain in WASP family proteins [139,145,146]. Directly transferring this result to cells is not trivial, as unbound G-actin might virtually be absent in cells, and most effects previously reported for CD (**2**) on tissue culture cells clearly cohere with its binding to F-actin. An example of counterintuitive cellular behavior was reported by Bear and colleagues (2002), who found that treatment of Rat2 cells with 25 nM CD (**2**) led to an increase in cell translocation rate, whereas usage of 500 nM CD (**2**) caused a significant decrease [101]. This demonstrates that concentration ranges exist in which F-actin and its regulatory network are maneuvered into a behavior that cannot be explained by the inhibition of active F-actin polymerization alone in a monocausal fashion (as intuitively, a decrease in active actin polymerization should reduce and not increase translocation speed). However, it is not difficult to imagine that this implies that cytochalasans could exert their effects on cytoskeletal dynamics beyond the mere disturbance of the F-actin network, or at least in ways that require careful re-consideration of the reasons for cellular phenomena, with more precise consequences yet to be characterized for the associated ABPs and cytoskeletal networks involved. Secondary binding sites possibly contributing to such observations will be covered in a later section below.

## 5. Cytochalasins and Reported Effects on Actin-Binding Proteins and Actin Architecture

Actin-binding proteins are capable of tightly controlling actin dynamics, maintaining the shape of cells, and mediating their motility. Hence, it appears sensible to study how actin-dependent structures, factors regulating them, and their dynamicity are affected by interference with actin polymerization by the presence of a given cytochalasin. The formerly introduced microspikes (termed filopodia in the neuronal growth cone), for instance, have been shown to vanish under acute treatment with CB (**1**) [147], probably due to quite selective inhibition of their elongation as compared to the rest of the growth cone, but how? An important gene family encoding proteins associated with microspike formation, as already mentioned above [110], is Ena/VASP (enabled/vasodilator stimulated phosphoprotein), known to support actin assembly in vitro and to accumulate at sites of active actin polymerization in cells, including the tips of filopodia and lamellipodia as well as focal adhesions [148]. It should also be remembered that Mena and VASP—prominent members of this family (see above)—were described as being displaced from protruding leading edges upon fixation and staining following treatment periods of 30–120 min with CD (25–150 nM, **2**), as documented by immunofluorescence. Potential technical problems arising from the indirect protein labeling with antibodies were excluded by confirming these observations with live-cell imaging with EGFP-tagged proteins and local treatment with CD (**2**; 0.5–1 µM 2–5 min), again leading to delocalization of Ena/VASP family members [101,122,149,150,151,152]. Ena/VASP delocalization in these conditions appears quite specific, as other lamellipodial proteins such as the prominent Arp 2/3 complex binding protein cortactin [153] did not display this behavior [152]. In contrast, local application of high doses (~52 µM needle concentration) of CB (**1**) at protruding mouse melanoma cells (B16-F1) seeded on a laminin-coated substrate led to a strong increase of the EGFP-VASP signal at the lamellipodial tip, suggesting a selective block of its dissociation from these sites [154] (and own unpublished data). A common mechanism for all cytochalasins in causing the delocalization of Ena/VASP proteins from protruding membranes can thus be excluded. Even if one considers the possibility that high concentrations of cytochalasins may cause increased nucleation of filaments—which remains to be shown in the intracellular environment—that would then be capable of recruiting more VASP, this scenario would not work easily because the high concentrations of CB (**1**) should at the same time outcompete VASP [88,133,155]. Moreover, an experiment using photoactivatable actin in BSC-1 cells in the presence of high concentrations of CD (**2**) did not lead to a detectable increase in nucleation or actin accumulation events that would support this hypothesis [128]. Clearly, additional experiments are required to clarify the exciting, differential effects on tightly connected subcellular structures (lamellipodial filaments vs. microspike bundles in the neural growth cone) or the currently conflicting observations concerning the impact on the subcellular positioning of Ena/VASP family proteins of cytochalasins.

Another interesting question is if cytochalasins might have actin isoform-specific effects. In total, there are six different actin isoforms present in mammalian cells: α-cardiac, skeletal, and smooth muscle actin (actC1, actA1, and actA2) mostly expressed in heart, skeletal and muscle tissue, respectively, and γ-cytosolic and γ-smooth muscle actin (actG1 and actG2), with the latter being predominant in smooth muscle tissue and the former together with β-actin (actB) ubiquitously expressed [156]. The isoforms differ only by a few amino acids in the N-terminus of the polypeptide sequence, but appear to have differential functions considering their expression patterns in different tissues. However, it is still not entirely clear if this is because of their biochemical properties or because of other factors, such as the selective binding of ABPs to specific actin isoforms. In addition, recent studies suggest that the reasons for at least organismal knockout phenotypes actually lie in the locus itself serving a regulatory purpose rather than the specific polypeptide it encodes for. Specifically, the embryonic lethal actB knockout phenotype in mice could be rescued by the actG1 sequence engineered into the *actB* gene locus [157]. However, there are also reports describing the selectivity of ABPs for given actin isoforms (actB is preferred over actG1 by DIAPH3 in the context of contractile ring formation), which have so far not been explained at the molecular level and harmonized with the physiological relevance mentioned above [158].

In protruding lamellipodia, the Arp 2/3 complex is crucial for creating a dense meshwork of F-actin, as it nucleates new daughter filaments after mother filament binding, being incorporated into the lamellipodial network in this process [64,159]. Previous work employing β- and γ-actin-specific antibodies suggested a partial preference for γ-actin incorporation in the lamella and less so in the lamellipodium, but an increased sensitivity of β-actin towards CD (**2**) [160]. Whether or not this differential sensitivity can be truly explained by the differences in actin isoforms at the protein level remains to be confirmed by independent methods. An alternative explanation for such isoform-specific effects might also be the preferential effects of cytochalasans on or interactions with ABPs. Notably, however, the literature also harbors reports on single-point mutations in the β-actin gene that decreased sensitivity, in this case in a tumor cell line called KB 3.1, which were enforced by chronic CB (**1**) exposure [44,161,162]. A mechanistic explanation for how these mutations would reduce cytochalasan’s mode of action is also still missing.

But back to the Arp 2/3 complex and structures that strictly depend on this complex, such as the lamellipodium [112,163,164]: In past decades, cytochalasins were successfully employed on highly dynamic, treadmilling actin networks at the cell periphery, foremost of all the neuronal growth cone [165] or the fish keratocyte lamellipodium [166]. Another Arp2/3 complex-dependent structure compromised in its formation by CD (**2**) constitutes the actin comet tail pushing vaccinia virus [167]. In the latter study, the drug was used as a tool to demonstrate the dependence—at the tail tip—of the turnover of the Arp2/3 complex activator N-WASP, essential for virus motility, on active actin polymerization. Irrespective of this conclusion, all these results suggest the impact of cytochalasins on dynamic actin remodeling in cells. Somewhat surprising, however, a recent study characterizing Arp2/3 complex-deficient fibroblasts described a highly reduced sensitivity in cells lacking this branching machine to at least low doses of CD (**2**) [168]. If correct, these results would imply the lack of capability of CD (**2**) to interfere with actin assembly by ABPs mediating Arp 2/3 complex-independent structures, a conclusion also put forward by the authors. Future studies will certainly unveil whether this notion holds true for distinct cytochalasans at full concentration ranges, but it would already be incompatible with the aforementioned selective abrogation of filopodial/microspike bundles in neuronal growth cones [147].

Aside from N-WASP, which is essential for Arp 2/3 complex activation at the surface of vaccinia viruses, the actin networks in lamellipodia are largely driven by a heteropentameric complex best known today as the WAVE regulatory complex (WRC) [129]. Interestingly, the WRC subunit Sra-1 (specifically Rac-associated-1) was recently reported to be significantly affected upon treatment with CD (**2**) [103]. Using superresolution microscopy and single particle tracking analyses, it was proposed that pushing or friction forces exerted by individual, growing actin filaments onto these complexes in the lamellipodium tip directly regulate their turnover at the plasma membrane. This conclusion was again consistent with the previously mentioned actin polymerization being capable of impacting the residence time of its regulators, measured here as arrest or at least slowdown of single particle movements along the membrane [103]. That such a behavior holds true for the entire complex and not only its individual subunits is suggested by the earlier description of Abi-1 forced to accumulate at the lamellipodium tip by CB (**1**) [154], which remains to be confirmed, also for the remaining subunits, in future studies. The hypothesis that WRC accumulation is solely caused by inhibition of actin polymerization and not necessarily by cytochalasin-specific interference with this process is supported by enrichment of the WRC subunit Hem-1 upon latrunculin B treatment at the leading edge and by its reduced turnover at this site [169]. Of note, however, is that such an effect of latrunculin has hitherto not been described for any other cell type or condition, at least to our knowledge, and for reasons that remain to be established. Notwithstanding this, active actin polymerization, at least in Arp 2/3 complex-dependent structures, appears to promote the turnover of its activators, i.e., WRC at the lamellipodium tip or N-WASP (and associated factors) at vaccinia virus surfaces (see above).

Another lamellipodial factor closely associated with the Arp 2/3 complex is CP. An interference of cytochalasans with CP and other proteins exerting a barbed end capping function had been discussed in a previous review, since all these actors are thought to bind to the F-actin barbed end [54]. Experiments reported so far focused on the CP subunit β2 and other factors capping activity was at least partially ascribed to, i.e., βcap73 [170], 88 K actin-binding protein [171], gelsolin [107], and tensin [172]. However, the capping activity of filament barbed ends does not always lead to blockage of filament elongation and, hence, short filament accumulation. Recently, for instance, a molecular mechanism has been suggested to harmonize the capping and nucleation-enhancing effects previously described for CP in Arp2/3-dependent actin networks with each other. In this mechanism, the protein binds to barbed ends and caps filaments, but it also acts in support of freeing NPFs such as N-WASP and WRC from previously bound filaments for new rounds of Arp2/3 complex activation. The protein thus serves an important role in the spatial nucleation of new actin filaments, allowing the productive growth and protrusion of lamellipodial networks [38,124,125]. The localization of CP-β2 in lamellipodia and motile actin-rich spots did not reportedly significantly change upon short-term cytochalasin treatments [173]. To what extent cytochalasins might directly or indirectly interfere with β-tentacle function in CP remains an open question [125].

Another study on an alternative, potential barbed end capper, βcap73, described a mere disruption of its barbed end binding to actin filaments by CD (**1**) [170,174]. Unfortunately, however, the physiological meaning of this observation has so far not been explored. Furthermore, Tensin-1 was found to be inhibited in phosphorylation in cells spreading in the presence of CD (**2**). However, the precise cause of this lack of phosphorylation or its consequences has hitherto not been investigated [25]. In contrast, the inhibition of actin polymerization affected by 88K-ABP was not altered in the presence of CB (**1**) [171], suggesting non-overlapping binding sites. A number of papers collectively found at least a partial reduction in sensitivity towards cytochalasins of actin filaments if associated with factors of the contractile machinery. These include F-actin pre-treated with meromyosin [87] and non-muscle tropomyosin—a protein thought to act as an actin scaffolding protein [175]. A similar conclusion was drawn from cells subjected to treatment with CD (**2**) and overexpressing the human heart LIM protein (hhLIM) [176].

Another lamellipodial tip compound studied is the protein lamellipodin (Lpd), thought to stabilize protrusions and optimize the formation of nascent adhesions [177]. It was found that the localization of Lpd did not change upon CD (**2**) treatment in the works of Krause et al. (2004) [150]. Conversely, Hansen and Mullins (2015) showed that a peptide derived from Lpd consisting of the actin-binding domain (ABD, amino acid positions 850–1250) was displaced from the lamellipodial edge by CD (**2**) [122]. This suggests that the interference of cytochalasins with the correct subcellular positioning of ABPs must involve additional parameters apart from just competing for the barbed end with the respective ABD.

Formins, some of which can accumulate at lamellipodial and filopodial tips [178], are *bona fide* barbed end binders that, as dimers, exhibit both actin filament nucleation and elongation capacities. In an initial study describing this fascinating activity, a fragment encompassing at least the FH1 (Formin-Homology-1) and FH2 domains of the yeast formin Bnip1 was blocked in actin assembly by CB (**1**). It was concluded that CB (**1**) interfered with the elongation but not the nucleation by the formin [102]. As mentioned above, however, it has so far not been finally settled if CB (**1**) can exert actin filament nucleation by itself, which might have contributed to the reported observations. Together, it will be essential in the future to systematically assess and compare how various cytochalasans can affect the biochemical activities of barbed end binders such as VASP, formins, CP, or additional ones mentioned below.

One additional interesting candidate is the severing and barbed-end capper gelsolin. Due to its barbed end capping activity, this protein was initially characterized as a factor promoting pointed end elongation [179]. Of note, the protein isolated from platelets in this manuscript and turned out to correspond to gelsolin, was concluded to exert effects highly comparable to CD (**2**). Furthermore, the addition of CB (**1**) to gelsolin-actin seeds showed no significant inhibition of elongation compared to gelsolin alone [102], indicative of gelsolin and CB (**2**) displaying overlapping binding sites. Importantly, this also confirmed that CB (**2**) does not interfere with filament elongation at the pointed end [102]. The actin-interacting protein 1 (Aip1) has been shown to carry out similar functions as gelsolin, which are the capping of barbed ends and the contribution to filament severing, albeit for Aip1 acting in concert with cofilin [180]. More specifically, this activity is exerted by a trimolecular complex between Aip1, cofilin, and coronin, as previously shown to operate, for instance, in the turnover of actin comet tails formed by *Listeria monocytogenes* [181]. In a follow-up study, Kueh and colleagues showed via single filament imaging experiments that CD (**2**), similar to CP, strongly inhibits disassembly by this trimolecular complex at both filament ends [128]. Interestingly, the authors proposed that this phenomenon, called bursting disassembly, occurs through a mechanism coined cooperative strand separation, which is both incompatible with and inhibited by barbed end binders such as CP and CD (**2**) [128]. Finally, CD (**2**) did not apparently interfere with severing mediated by cofilin alone.

Severing by cofilin occurs at transition zones between actin filament segments lacking or homogeneously decorated by cofilin [134]. Cofilin, upon F-actin binding, induces a twist, creating tension at aforementioned transition zones and causing local filament breakage. Interestingly, this mechanism is inhibited upon binding another F-actin-binding and stabilizing molecule derived from fungi, phalloidin [182]. Regardless of this, aside from F-actin, cofilin can also bind to actin monomers, which, at least *in vitro*, was reported to promote nucleation [183]. As opposed to the conclusion drawn by Kueh et al. (2008), a more recent study described the interference of CD (**2**) with cofilin binding to both G- and F-actin [184]. The authors concluded from co-sedimentation assays and using photoactivatable actin in live cell imaging experiments that CD (**2**) significantly reduced the amount of cofilin bound to F-and G-actin and that it inhibits both F-actin polymerization and depolymerization in living cells. Thus, they suggested that, among other effects, cofilin-dependent disassembly of F-actin is impeded by the cytochalasin and that the mechanisms behind the cytochalasans’ impact on the cytoskeleton are manifold. How all this is compatible with earlier observations of increased active cofilin levels upon CD (**2**) treatment in cardiomyocytes is currently unclear [23]. Clearly, more experiments are needed to clarify these discrepancies.

All aforementioned components and their responses to cytochalasans can be viewed in light of the fact that they operate in highly dynamic actin remodeling. Without wanting to imply that cytochalasans will not have any function on more static actin structures, the literature harbors indications that the latter structures are frequently less sensitive, or at least less severely compromised. A prominent example of this could be myosin II, a motor protein forming highly organized, prominent bundles in muscle and smaller analogous arrays in non-muscle cells, co-localizing with and exerting contractile functions in actin bundles and stress fibers. In neuronal growth cones, myosin II also contributes to actin retrograde flow, the process of the actin network moving from sites of actin filament assembly at the cell periphery towards the cell center, which remained largely unaffected by CB (**1**), consistent with an unaltered localization pattern in this region [147]. The latter observation is also consistent with the observation of continuous nuclear translocation in migrating keratocytes upon cytochalasan treatment, even when cellular movement, i.e., lamellipodial protrusion, is completely stopped. This nuclear translocation is considered to be dependent on overall cell shape and tension mediated by myosin II [166].

Another group of ABPs is known for operating below the plasma membrane, particularly at the cell cortex and/or connecting actin filaments, the spectrin superfamily [185,186]. Prominent members include spectrin and its largely ubiquitous isoform, fodrin, as well as ERM (ezrin, radixin, and moesin). Notably, binding to β-actin-sepharose of β-spectrin could be inhibited by CD (**2**) in vitro [174], a finding later rationalized in silico to possibly derive from an affinity, at least of CB (**1**), for the ABD of spectrin [187]. Fluorescence quenching experiments for the isolated ABD did indeed support this idea, although full-length spectrin exhibited a 10-times lower affinity for CB (**1**) binding [187]. The relevance of these findings in the cellular environment is unclear, but an expression study on the spectrin family members spectrin, ezrin, moesin, radixin, and fodrin in *Xenopus* 2F3 cells revealed these cells to react to the presence of CE (**55**) with decreased fodrin expression [22]. Whether or not the latter observation involves a direct fodrin–CE interaction remains to be established.

Two further ABPs have been studied in the context of actin inhibitory drugs, displaying either differential or congruent effects. The neuron-specific neurabin II, specifically associated with sites of dynamic actin remodeling such as spines, was shown to stay associated with these structures in the presence of CD (**2**), but to relocalize to the cytosol upon latrunculin B treatment [188]. In contrast, the Src kinase substrate and adhesion protein AFAP (actin filament-associated protein) was shown to be dissociated from F-actin upon treatment with both CD (**2**) and latrunculin A in this case, suggesting that its relocalization is tightly connected to the lack of actin filaments irrespective of the mechanism of the provoked filament loss [189].

Again, all these examples illustrate the complexity and diversity of effects on both actin turnover and actin regulators of the seemingly highly related cytochalasin-type drugs spotlighted here. A comprehensive list of the aforementioned proteins, including their behavior upon cytochalasan treatments, can be found in Table 1.

In the following, we aim to briefly present our thoughts on the common modes of treatment and concentration ranges found in the literature, and on working hypotheses for how cytochalasans might be developed into future anti-infectives. In other words, what is the true potential of cytochalasans? Should we consider them solely as tools for the dissection of cell biological phenomena, or what is their realistic potential for being employed in pharmaceutical or perhaps even clinical applications in the future?

An interesting view on the bioactivity of CD (**2**) in comparison to latrunculin B—differing from the barbed-end elongation interference mode of action by G-actin sequestration—had been shared by Wakatsuki et al. (2001) [137]. Here, the authors sought to explore the discrepancy between concentration ranges used in CD (**2**) treatments in vitro vs. *in cellulo*. Specifically, they emphasized that the dissociation constant of CD (**2**, 2 nM) bound to isolated F-actin in biochemical experiments is orders of magnitude lower than the concentrations applied to achieve total filament network disruption in cells (1–10 µM) [54]. To examine more carefully the effects of lower CD (**2**) concentrations on cells, they explored the mechanical properties of fibroblasts treated with varying concentrations of CD (**2**). This indeed revealed that cellular dynamic stiffness and exhibited contractile force were affected at concentrations as low as 2 nM, although changes visible by immunofluorescence started to occur at ten times higher concentrations (20 nM). Such modest effects started to manifest as small F-actin aggregates, which culminated in a fully disrupted actin filament network at concentrations of 2 µM, virtually eliminating cellular dynamic stiffness. The concentration inducing half-maximal effects for actin cytoskeletal disruption and mechanical parameters was estimated to be 0.25 µM, which is still orders of magnitude higher than the concentrations used to inhibit actin polymerization in vitro [54]. Latrunculin B’s effective concentration range, however, only spanned from 160–630 nM. Interestingly, Wakatsuki and colleagues thus hypothesized (also based on mathematical simulations) that this difference in CD (**2**) exhibiting increasingly dramatic effects on actin filament organization over a larger concentration range is due to the interference of cytochalasins not just with actin, but also with at least two other barbed-end capping proteins that CD (**2**) is proposed to compete with [137].

As elaborated on in detail above, cytochalasans are commonly used in the literature as tools to depolymerize actin filaments, with the assumption that at sufficiently high concentrations, this depolymerization is still specific and virtually complete. This, however, is not nearly as trivial as commonly assumed. With DNase I-actin binding assays, Howard and Lin (1979) did indeed show that CD (**2**) inhibits actin filament polymerization, but also that this coincides with increased levels of unpolymerized G-actin in platelets, likely caused by a shift of filament kinetics towards depolymerization [90], later also reported for different cell lines by Schwingshackl et al. (2015) [195]. In direct contrast to these findings, other authors [184,196] could not observe net changes in G- to F-actin ratio after treatment with CD (**2**). This was explained by the reduction in individual actin filament length being compensated by an increase in filament numbers so that total filament mass would not change (also discussed by [197]). The nucleation and polymerization of a plethora of new, shorter filaments caused by cytochalasan treatment, however, has, to the best of our knowledge, not unequivocally been demonstrated in living cells. The interpretation that cytochalasans solely effect intracellular F-actin depolymerization is further complicated by the previously discussed potential sensitivity of cofilin or cofilin-containing actin depolymerization complexes to these drugs. That this also extends to additional ABPs is demonstrated by a study on α-crystallin, a chaperone, which was shown to protect filamentous actin against depolymerization induced by CD (**2**) in a phosphorylation-dependent manner [190]. Finally, it should be mentioned that a recent report showed that manipulations with cytoskeletal drugs drastically change the biophysical environment of cells. CD (**2**), for example, has been shown to reduce the extent of “molecular crowding” in cells, while also reducing the volume of U-2 OS cells by up to 25% [198]. This, in our understanding, may lead to rather unusual intracellular conditions from the perspective of ABPs, i.e., non-physiological compaction of F-actin, which might trap and thus inactivate, e.g., lamellipodial proteins. All these considerations confirm that we are in need of a more systematic side-by-side comparison of the precise effects of various cytochalasans, using a range of experimental approaches and cellular systems.

What has so far not been conclusively addressed, neither in the literature nor by ourselves up to this stage, is how chemical differences in cytochalasan structure or cellular properties impact on the effectivity of cytochalasan’s mode of action. This also includes parameters such as, e.g., plasma membrane permeability. More specifically, if developing cytochalasan libraries used in screens for potential novel activities being exploited in the context of treatment against infectious disease symptoms or otherwise diseased cells (e.g., tumor cells), a key parameter not to be underestimated will be making the cytochalasan accessible to the target. In the case of losing activity, the reduction in affinity for the wanted target will only be one possible explanation; parameters such as stability, solubility in aqueous solutions, plasma membrane permeability, or even diffusion throughout the extremely crowded cytosol of cells will be equally relevant to be considered. In an ideal world, a future cytochalasan will be developed, freely diffusing to and trespassing the plasma membranes of cells harboring its specific target, whether it be the barbed end of a subset of actin filaments or even a specific ABP.

## 6. Potential Non-Barbed End Binding Activities of Cytochalasans in Actin-Dependent Model Systems

Earlier in this review, we mentioned that the effective concentrations of cytochalasans to exert changes on the F-actin network in tissue culture cells are much higher than one would assume, given how potently cytochalasans inhibit polymerization of purified actin in biochemical experiments. While the total number of barbed ends in cells is unknown, secondary binding targets may titrate the effective intracellular cytochalasan concentration available. Non-actin targets were described for CB (**1**) rather early, such as high-affinity binding sites for glucose transporters [80,81,199,200], interfering with the transport of glucose and other hexoses [199], or with the function of transmembrane channels. For instance, the hKv1.5 channel is targeted by CA (**3**) and CB (**1**), probably due to their structural similarity. This process has been concluded to be actin-independent, since CD (**2**), CJ (**58**) and the actin-stabilizing fungal toxin phalloidin [54] were only weakly or not at all able to inhibit the channel in the concentration ranges tested [21]. In the context of glucose transporters, a recent paper proposed that the activity of cytochalasans is mediated not only by the phenylalanine amide moiety present in CB (**1**), but also by other synthetic compounds otherwise non-related to the cytochalasan backbone [15]. This observation would suggest that glucose transporter inhibition should not be restricted to CB (**1**), but extend to various cytochalasans harboring this moiety, including CD (**2**). This is inconsistent, however, with a much earlier study in which SAR on twenty cytochalasans had been compared in erythrocyte ghosts [80]. The authors had shown, first of all, that not all phenylalanine amide moiety-containing cytochalasins are acting in an inhibitory fashion. Furthermore, those cytochalasans that were active included not only CA (**3**) and CB (**2**), but also chaetoglobosins, which lack this moiety. The reason why ChgB, E, and F (**5**, **7**, **8**) all inhibit glucose transport is currently unclear, as the binding site considered crucial for inhibition is bound with high affinity, but shows lower effects on glucose transport when compared to CA (**3**) and CB (**1**) [81]. This notwithstanding, the fact that a subset of cytochalasans inhibits actin assembly, but not glucose transport (CC-CH, [80]) constitutes a prominent example of non-actin-related cytochalasan targets.

**Figure 14:**
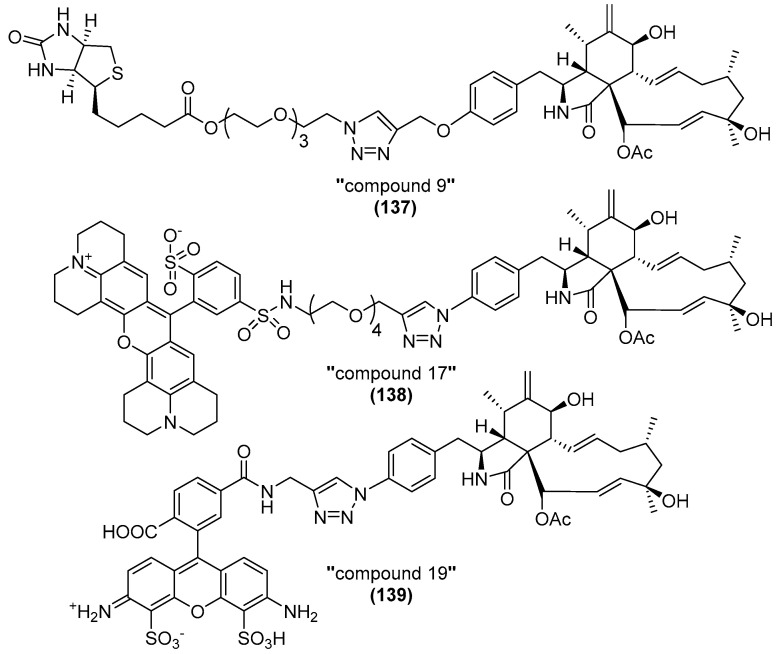
The biotin (“compound 9”, **137**), Texas Red (“compound 17”, **138**) and Alexa 488 (“compound 19”, **139**)-linked pyrichalasin H (**91**) derivatives generated by click-chemistry following a mutasynthetic approach presented by Wang et al. (2020) [202].

Notably, in a more recent study from our lab with a functionalized pyrichalasin H (**91**) used for actin filament staining in cells (“compound 17”, **138**, see Figure 14), we found staining patterns not necessarily expected from just staining filament barbed ends. Although in this study, we have interpreted the staining pattern as an apparent, more homogeneous distribution of barbed ends throughout actin structures in cells, a binding activity of this cytochalasin-derivative apart from the barbed end, for instance along the filament, could not be entirely excluded.

The idea of cytochalasins potentially binding to the side of filaments is supported at least in part by previous studies. In 1979, for instance, Hartwig and Stossel already concluded that CB (**1**) possessed two binding sites for F-actin; one high-affinity binding site (K_a_ of 10^8^ M^−1^) and a low-affinity one (K_a_ value ≤ 2 × 10^6^ M^−1^) [136], although they did not distinguish in this case between distinct interactions along the filament. Wang et al. (1990), however, concluded that filament side binding at exceedingly high concentrations of CB (**1**) to actin imposed conformational changes on those filaments, ultimately causing the loss of high-affinity CB (**1**) binding to their barbed ends [201]. Foissner et al. (2007) noted that CD (**2**), but not latrunculin A, disrupted the myosin-dependent motility of the characean alga *Nitella pseudoflabellata,* possibly through filament side binding [192]. Finally, Sampath and Pollard in the early 1990s also interpreted their actin filament elongation assays to suggest that cytochalasans can, at least with increasingly reduced affinity, bind further distal to the filament end [11].

The functionalized pyrichalasin H (**91**) derivatives, in particular the fluorescently labeled one (**138**), harbor the potential to characterize less clearly established binding modes to filaments, for instance, using single filament total internal reflection fluorescence microscopy (TIRF-M) experiments or perhaps time-lapse compatible superresolution approaches in the future. This also holds true for the characterization of the direct or indirect effects of cytochalasans on different ABPs (see above). The mutasynthesis described to produce semi-synthetically linkable pyrichalasin H (**91**) could in the future be adapted to generate new probes to, e.g., fish for secondary targets through the addition of other functional groups, as exemplified by the already synthesized, biotin-linked cytochalasin H (“compound 9”, **137**) [202].

## 7. Conclusions

The usage of cytochalasans as actin inhibitory tool compounds in research over the last decades in cell biology led to an extensive accumulation of data related to the biochemical and molecular aspects of their interaction with actin, as well as the effects exhibited by these compounds on specific actin structures. Aside from these aspects, even actin-independent activities have been discovered and characterized to a certain extent, but they are certainly not comparable yet to aforementioned activities. In spite of detailed characterizations of the consequences of cytochalasan treatments for distinct actin structures, we would like to emphasize that interpretations of the precise molecular reasons for observed phenotypes are as complex as the actin structures themselves. In other words, we propose that we urgently need to improve our molecular understanding of the cytochalasans’ mode of action before we seriously start considering employing them as fully reliable, robust tools for research or even therapeutics. Of note, the vast majority of literature was generated using two of the most prominent members of this compound family, CB (**1**) and CD (**2**), without exploiting the huge chemical diversity that comes along with the now more than 400 naturally occurring unique substances. Admittedly, SAR studies on a limited set of distinct cytochalasans have recently been published, but a precise definition of the core structure responsible for actin inhibitory function and a full molecular understanding of reversibility vs. irreversibility of effects are still lacking. Furthermore, serving as a single example for illustrating the problems arising from ignoring chemical differences, CB (**1**) and CD (**2**) have traditionally been used in the literature in an interchangeable fashion. As elaborated on above, this led to virtually unresolvable problems concerning correct interpretations of the effects of inhibiting actin polymerization on the subcellular positioning and dynamics of some actin polymerization regulators, such as VASP (see above). Finally, if considering this fascinating group of compounds as potential future therapeutic agents, we will—aside from solving all aforementioned problems—have to address issues such as cell- or tissue-specific targeting to potential sites of action, physicochemical features such as solubility and membrane permeability, as well as availability.

## Figures and Tables

**Table 1:** Summary of studies reporting effects of different cytochalasans on actin-binding and non-actin-binding proteins, together with reported treatment duration and experimental methodology employed. Due to the complexity and multiplicity of the results summarized here, we strongly recommend readers to review the original works for context.

Protein	Cytochalasan	Dosage	Treatment Duration	Experiment	Observed Effect	Source
**Arp2/3 complex**						
Arp3	CD (**2**)	0.1 µM	30 min	Immunofluorescence	Localization unaffected	[160]
p34Arc (Arp2/3)	CD	0.1 µM	30 min	Immunofluorescence	Localization unaffected	[160]
**Capping proteins**						
88 K actin binding protein (capping protein)	CB (**1**)	0.1–2.5 µM		Viscometry, light scattering, A_237_ measurement	No inhibition by CB, but 88K inhibits CB effect	[171]
CP-β2 (mouse capping protein)	CD	1 µM	2 min	Live cell imaging of GFP-tagged CP-β2	No effect on CP-β2 distribution	[173]
Gelsolin	CB	2 µM		Pyrene assay	Negligible effect on gelsolin-bound filaments	[102]
Tensin (crosslinking and capping protein)	CD	1.5 µg/mL	30 min	Immunoprecipitation	Tyrosine phosphorylation inhibited	[25]
βcap73 (capping protein)	CD	10 nM- 15 µg/mL		Actin affinity matrices	Binding to β-actin largely inhibited	[170]
**Ena/VASP family proteins**						
Mena	CD	25–150 nM	5–30 min	Immunofluorescence, live cell imaging	Delocalization	[101,150,152]
VASP	CB	52 µM (in needle)	2 min	Live cell imaging	Accumulation	[154]
VASP	CD	25 nM–1 µM	2 min–2 h	Immunofluorescence, live cell imaging	Delocalization	[101,122,149,150,151,152]
**Ezrin/radixin/moesin (ERM) family proteins**						
Ezrin	CE (**55**)	1.5 µM	60 min	Gradient centrifugation of treated cell lysate	Protein abundance unaffected	[22]
Ezrin	CD	2 µM- 15 µg/mL		Actin affinity matrices	CD competes with binding on actin affinity matrices	[170,174]
Radixin	CE	1.5 µM	60 min	Gradient centrifugation of treated cell lysate	Protein abundance unaffected	[22]
Fodrin	CE	1.5 µM	60 min	Gradient centrifugation of treated cell lysate	Protein abundance decreased	[22]
Moesin	CE	1.5 µM	60 min	Gradient centrifugation of treated cell lysate	Protein abundance unaffected	[22]
**Proteins examined in context of *L. monocytogenes*** **actin comet tails**						
Cofilin, Coronin, Aip1	CD	Concentration titration 0–3 µM		Single filament imaging	Activity on F-actin disassembly inhibited	[128]
**Myosins**						
Heavy meromyosin	CB	~100–420 µM		ATPase activity assay	ATPase activity inhibited	[9]
Heavy meromyosin (HMM)	ChJ (**54**)	0.71 µM	5 min	Electron microscopy of HMM-decorated F-actin	Binding to F-actin unaffected, but suppresses ChJ-mediated depolymerization	[87]
Myosin II	CB	3–57 µM	20 min	Viscometry	Competition of myosin II and CB for actin binding	[9]
Myosin II	ChJ	2 mol per mol actin	5 min	ATPase activity assay	ATPase activity reduced	[87]
Myosin II	CB	350 nM		Live cell imaging	Localization unaffected	[147]
**Other ABPs**						
AFAP (actin filament-associated protein)	CD			Gradient centrifugation	Increased in cytosolic fraction and reduced in cytoskeletal fraction	[189]
Bn1pFH1FH2	CB	2 µM		Pyrene assay	Partial inhibition of filament elongation, but not nucleation	[102]
Cofilin	CD	0.3–10 µM	10–120 min	*In vitro* sedimentation and BiFC assay	Binding to G-and F-actin inhibited	[184]
Cofilin	CD	5–10 µM	10 min	Western blots of whole cell lysates and comparison of phosphorylation status	Activated via dephosphorylation	[23]
Cortactin	CD	25 nM	30 min	Immunofluorescence	Localization unaffected	[152]
α-Crystallin	CD	1 or 2 mol per mol actin	35 min–20 h	Pyrene assay	CD-mediated depolymerization alleviated by α-crystallin	[190]
hhLIM (novel human heart LIM protein)	CB	?	10–120 min, 30 min	Live cell imaging, Immunoprecipitation	CB-mediated inhibition of polymerization and induced depolymerization alleviated by hhLIM	[176]
Lpd	CD	150 nM	30 min	Immunofluorescence	Localization unaffected	[150]
Lpd^850–1250aa^ (ABD of Lpd)	CD	100 nM	~2 min	Live cell imaging	Delocalization	[122]
Neurabin-II (spinophilin)	CD	10 nM–2 µM	20 min–2 h	Immunofluorescence and live cell imaging	No effect on association with F-actin	[188]
N-WASP	CD	10–200 nM	30 min	Immunofluorescence	Localization unaffected	[101]
Profilin	CD	?	?	?	Competitive binding of profilin and CD to Mg-ATP-actin	[191], Discussed by [192]
Spectrin	CE	1.5 µM	60 min	Gradient centrifugation of treated cell lysate	Protein abundance unaffected	[22]
Spectrin	CB	2 µM		Pyrene assay	Lack of effect on inhibition of F-actin polymerization	[103]
Spectrin	CB	2–60 µM	Steady-state titration	*In vitro* fluorescence quenching, in silico computational modeling and docking	CB binding with moderate affinity	[187]
ABD of spectrin	CB	2–60 µM	Steady-state titration	*In vitro* fluorescence quenching, in silico computational modeling and docking	10× increase of affinity to CB as compared to spectrin	[187]
Tropomyosin	CD, CB	2–20 µM, 100× higher		Viscometry	Protection of F-actin against CB and CD upon saturated binding	[175]
**Proteins examined in context of** **Vaccinia virus actin tails**						
ARPC5	CD	1 µM	Immediate, 30 min	Live cell imaging with FRAP, Immunofluorescence	Reduction of turnover at vaccinia virus actin tails	[167]
GRB2 [154])	CD	1 µM	Immediate, 30 min	Live cell imaging with FRAP, Immunofluorescence	Unaffected turnover at vaccinia virus actin tails	[167]
Nck	CD	1 µM	Immediate, 30 min	Live cell imaging with FRAP, Immunofluorescence	Unaffected turnover at vaccinia virus actin tails	[167]
N-WASP	CD	1 µM	30 min	Live cell imaging	Reduction of turnover at vaccinia virus actin tails	[167]
WIP	CD	1 µM	Immediate, 30 min	Live cell imaging with FRAP, Immunofluorescence	Reduction of turnover at vaccinia virus actin tails	[167]
**WAVE and WAVE regulatory complex (WRC)**						
Abi-1	CB			Live cell imaging	Accumulation	Unpublished [154]
Sra1	CD	0.1–1 µM	~1–20 min	Live cell imaging	Reduction of turnover at and translocation along leading edge	[103]
**Non-actin-related targets**						
Glucose transport through erythrocyte membranes	CA (**3**), CB, CB-7-monoacetate, 24-deoxaphomin, proxiphomin, protophomin, ChgB (**5**), ChgE (**7**), ChgF (**8**)				Inhibition of glucose transport	[80]
hGLUT1	CB	1 mM to 10 mg/mL of purified protein		Co-crystallization	CB binding to the central cavity of hGLUT1	[15]
GLUT1-4	CB				Inhibition of glucose transport	[193,194]
hKv1.5 channel	CA (**3**) CB, CD, CJ (**58**)				Reduced activity upon CA and CB, less prominent effect upon CD and CJ	[21]
**Further actin-associated effects**						
ENaC	CE CD	1.5 µM	15–60 min	Single channel patch clamp experiments, immunoprecipitation	Decrease of amiloride-sensitive transepithelial current, reduced interaction with MARCKS	[22]
GLUT4	CD				Prevention of insulin induced translocation	[24]
L-type Ca^2+^ channel	CD	5–10 µM	5–10 min	Measurement of whole-cell currents	Phalloidin-preventable reduction of Ca^2+^ currents in guinea pig cardiomyocytes	[23]

## Data Availability

Not applicable.
