# Correction: Lambert et al. Cytochalasans and Their Impact on Actin Filament Remodeling. *Biomolecules* 2023, *13*, 1247

**DOI:** 10.3390/biom15071030

**Published:** 2025-07-16

**Authors:** Christopher Lambert, Katharina Schmidt, Marius Karger, Marc Stadler, Theresia E. B. Stradal, Klemens Rottner

**Affiliations:** 1 Molecular Cell Biology Group, Helmholtz Centre for Infection Research (HZI), Inhoffenstrasse 7, 38124 Braunschweig, Germany; 2 Department of Cell Biology, Helmholtz Centre for Infection Research (HZI), Inhoffenstrasse 7, 38124 Braunschweig, Germany; 3 Department of Microbial Drugs, Helmholtz Centre for Infection Research and German Centre for Infection Research (DZIF), Partner Site Hannover/Braunschweig, Inhoffenstrasse 7, 38124 Braunschweig, Germany; marc.stadler@helmholtz-hzi.de; 4 Division of Molecular Cell Biology, Zoological Institute, Technische Universität Braunschweig, Spielmannstrasse 7, 38106 Braunschweig, Germany; 5 Braunschweig Integrated Centre of Systems Biology (BRICS), 38106 Braunschweig, Germany

In the original publication [1], there were some mistakes in Figures 2–12 and Scheme 1 as published. The mistakes appear in the chemical structures, majorly concerning the stereochemistry of a number of distinct structures. The corrected Figure 2, Figure 3, Figure 4, Figure 5, Figure 6, Figure 7, Figure 8, Figure 9, Figure 10, Figure 11 and Figure 12 and Scheme 1 appear below. The authors state that the scientific conclusions are unaffected. This correction was approved by the Academic Editor. The original publication has also been updated.

## Figures and Tables

**Figure 2:**
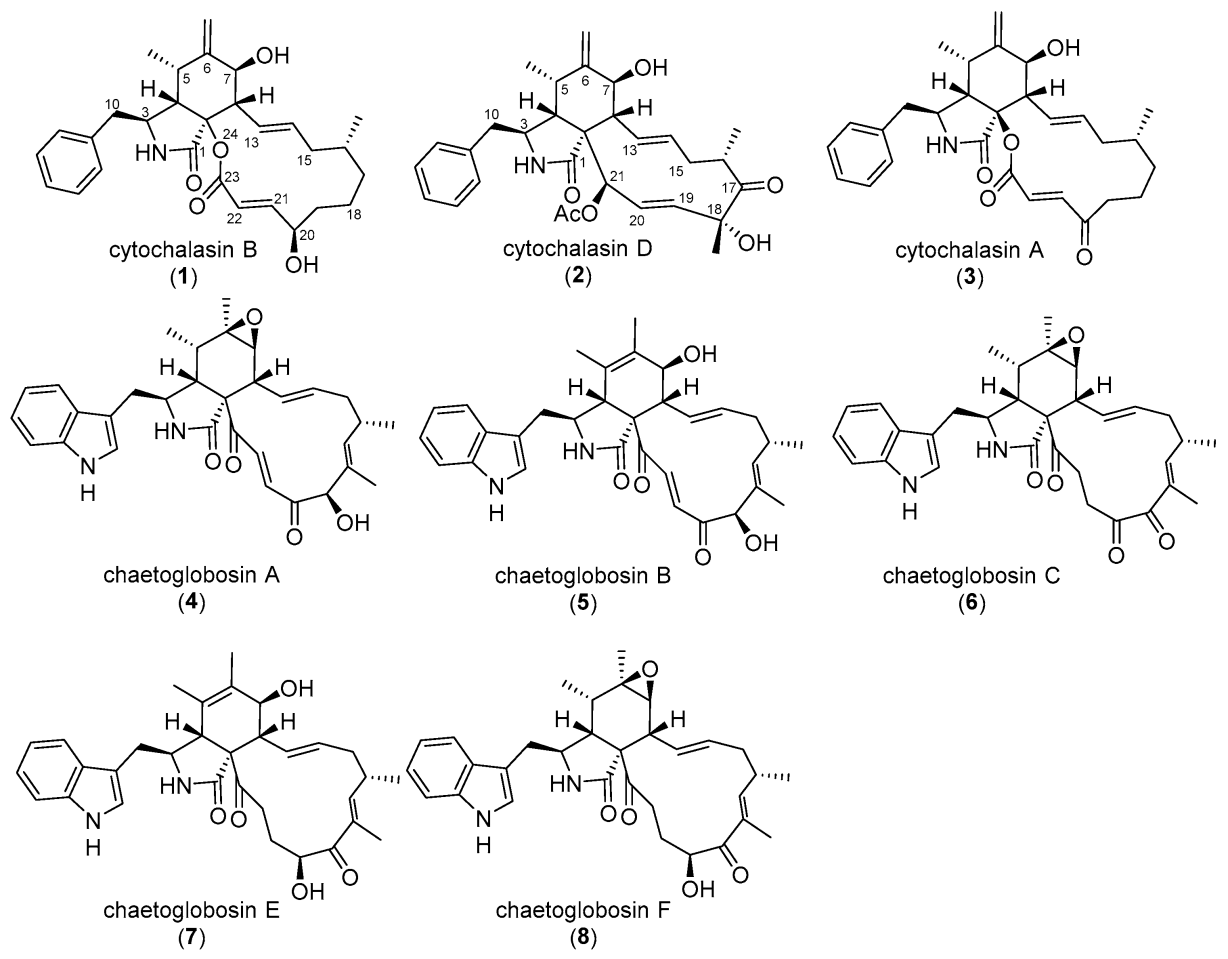
Structures of cytochalasin B (CB, **1**), D (CD, **2**), A (CA, **3**), and chaetoglobosin A-F (ChA-F, **4**–**8**).

**Figure 3:**
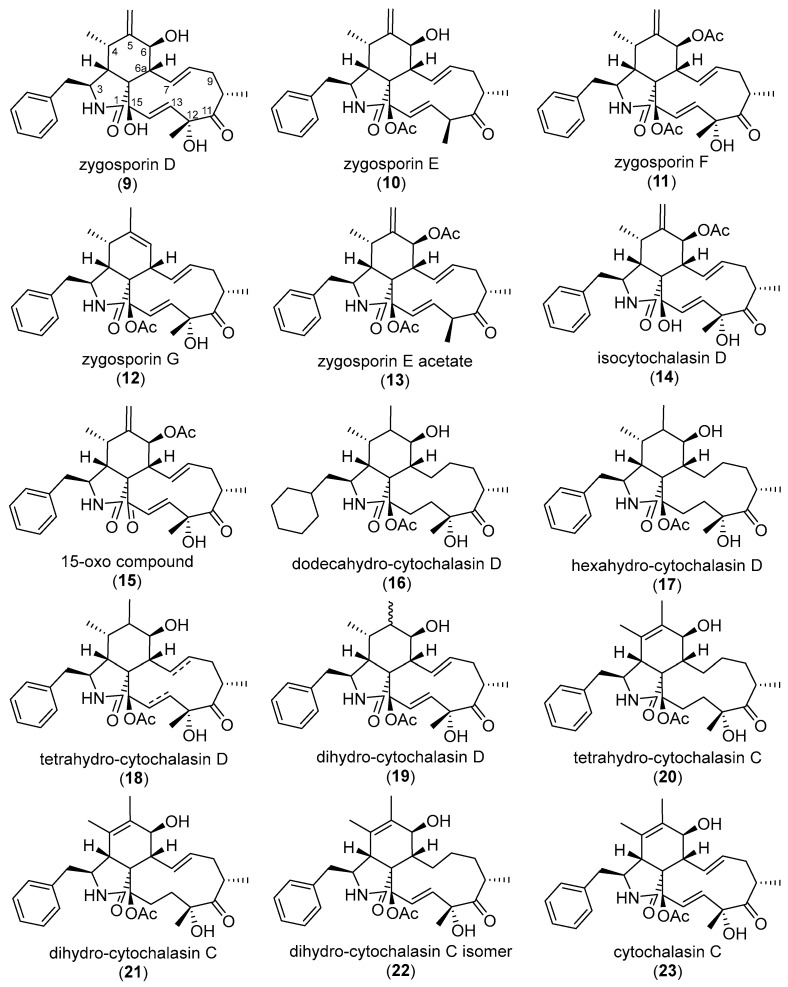

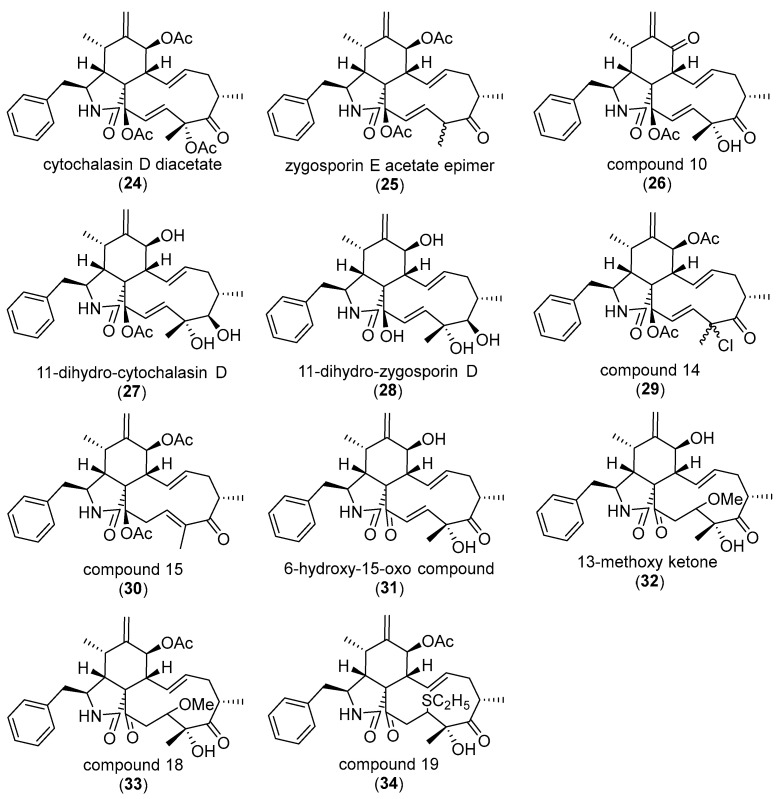

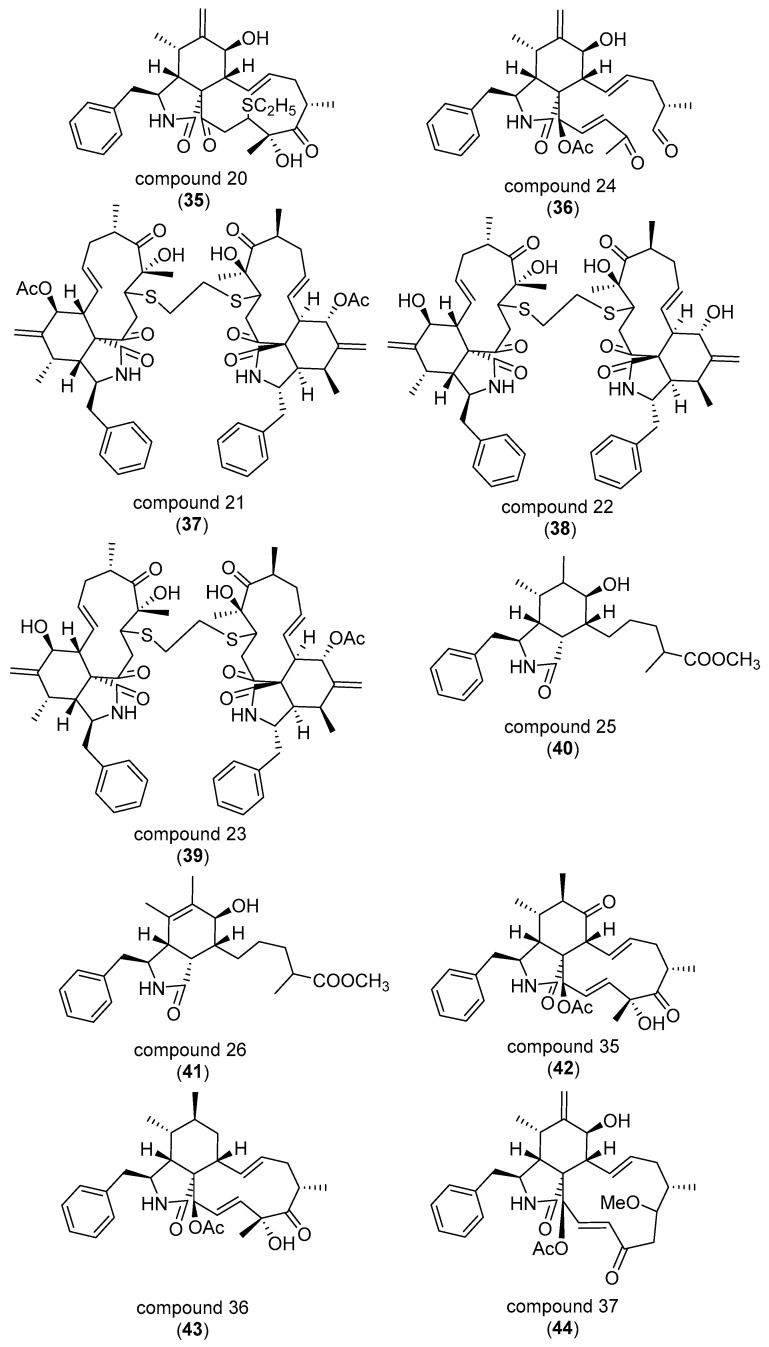
Structures of cytochalasin D derivatives (**9**–**44**) tested by Minato et al. (1978) [56].

**Figure 4:**
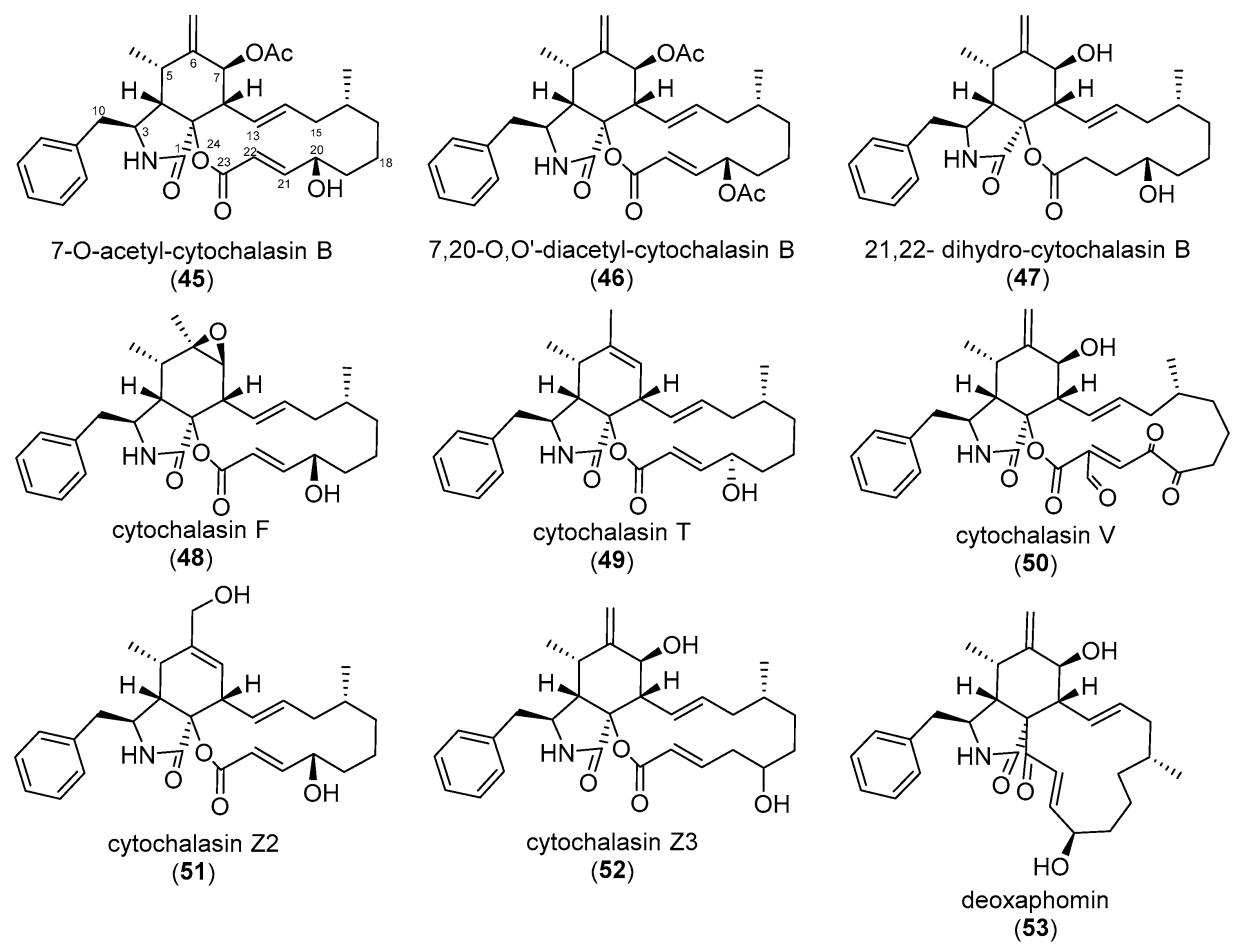
Structures of cytochalasans (**45**–**53**) investigated by van Goietsenoven et al. (2011) [58].

**Figure 5:**
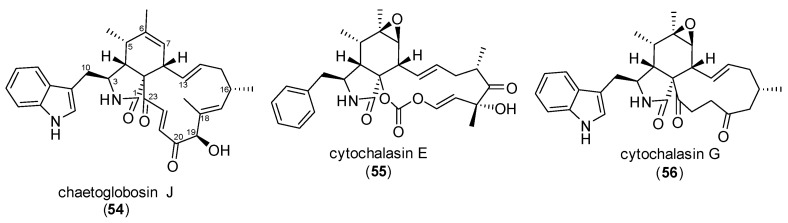
Structures of cytochalasans (**54**–**56**) tested by Löw et al. (1979) [67].

**Figure 6:**
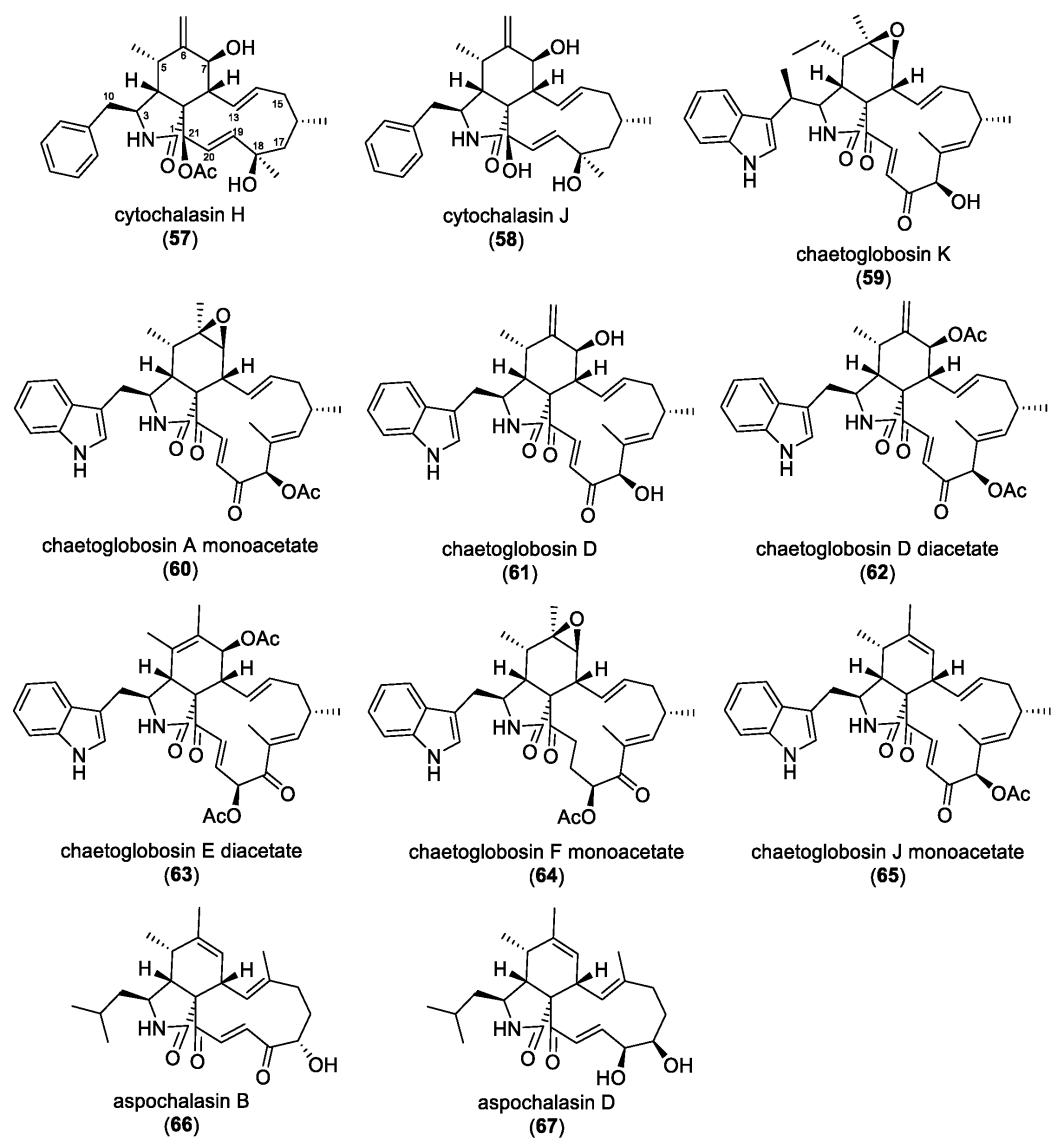
Structures of cytochalasans (**57**–**67**) tested by Yahara et al. (1982) [69]. The following compound names were interchangeably used by Sekita et al. (1985): 19-O-acetyl-chaetoglobosin A (**60**); 7,19-O-diacetyl-chaetoglobosin D (**62**); 7,19-O-diacetyl-chaetoglobosin E (**63**); 19-O-acetyl-chaetoglobosin F (**64**); 19-O-acetyl-chaetoglobosin J (**65**) [57].

**Figure 7:**
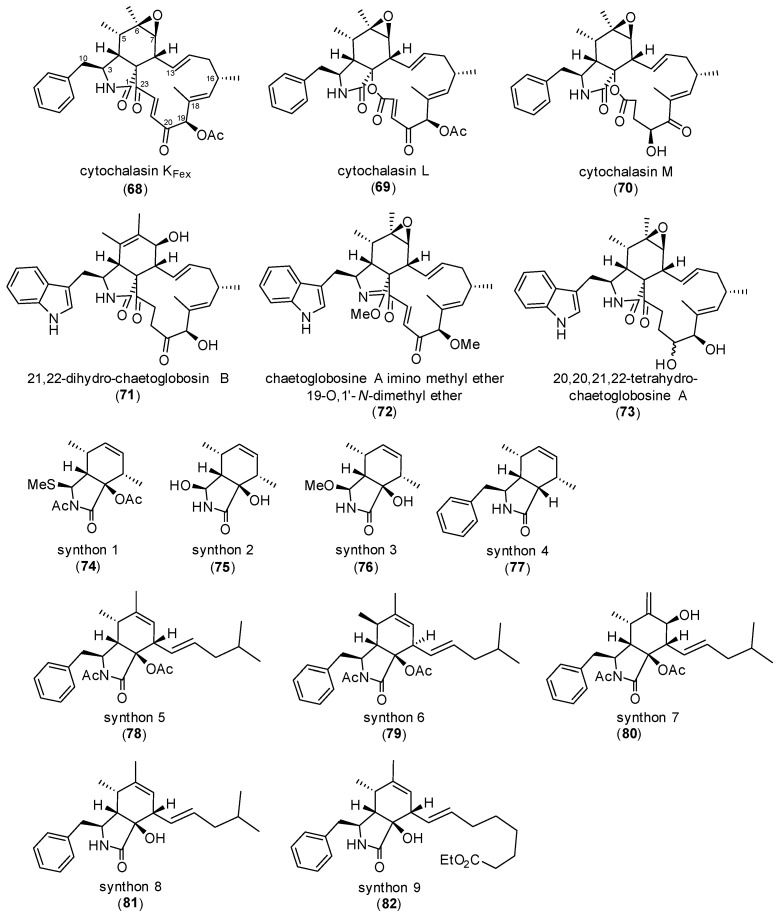
Structures of cytochalasans (**68**–**73**) and cytochalasin B synthones (**74**–**82**) analyzed by Sekita et al. (1985) [57].

**Figure 8:**
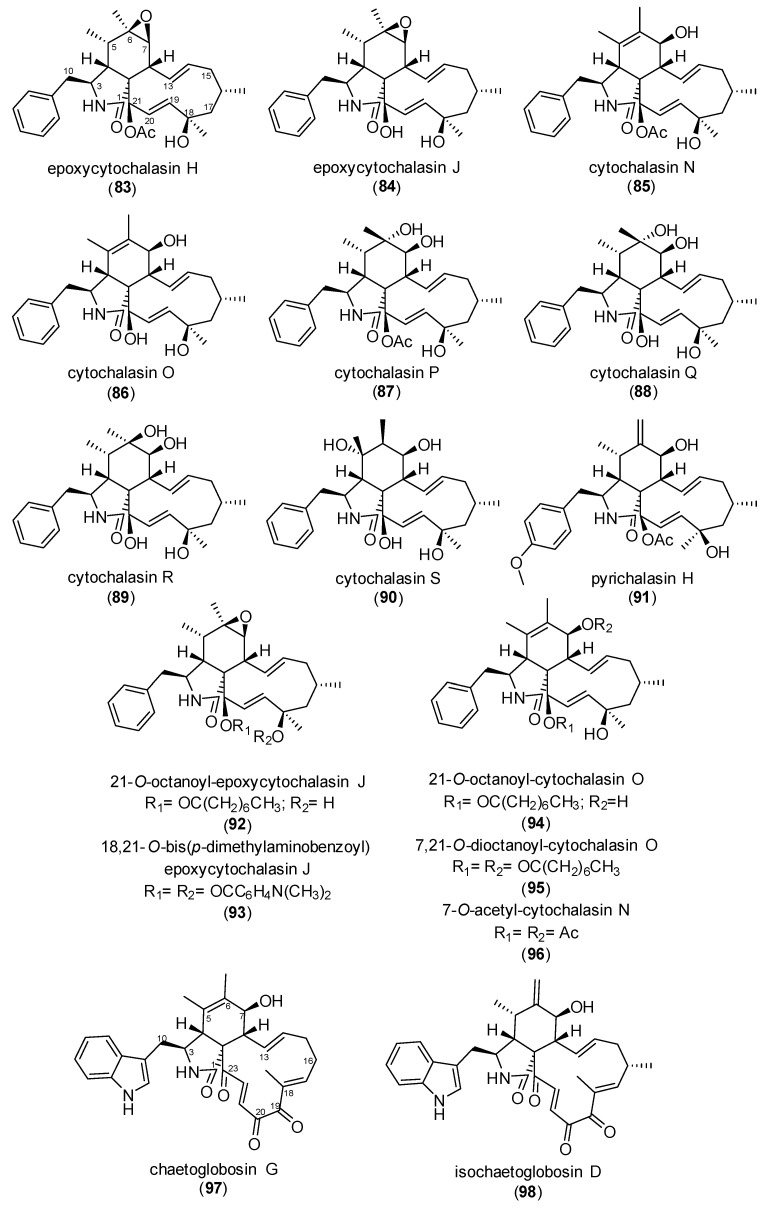
Structures of cytochalasans (**83**–**96**) tested by Hirose et al. (1985) [85] and chaetoglobosin G (**97**) and isochaetoglobosin D (**98**) by Thohinung et al., 2010 [92].

**Figure 9:**
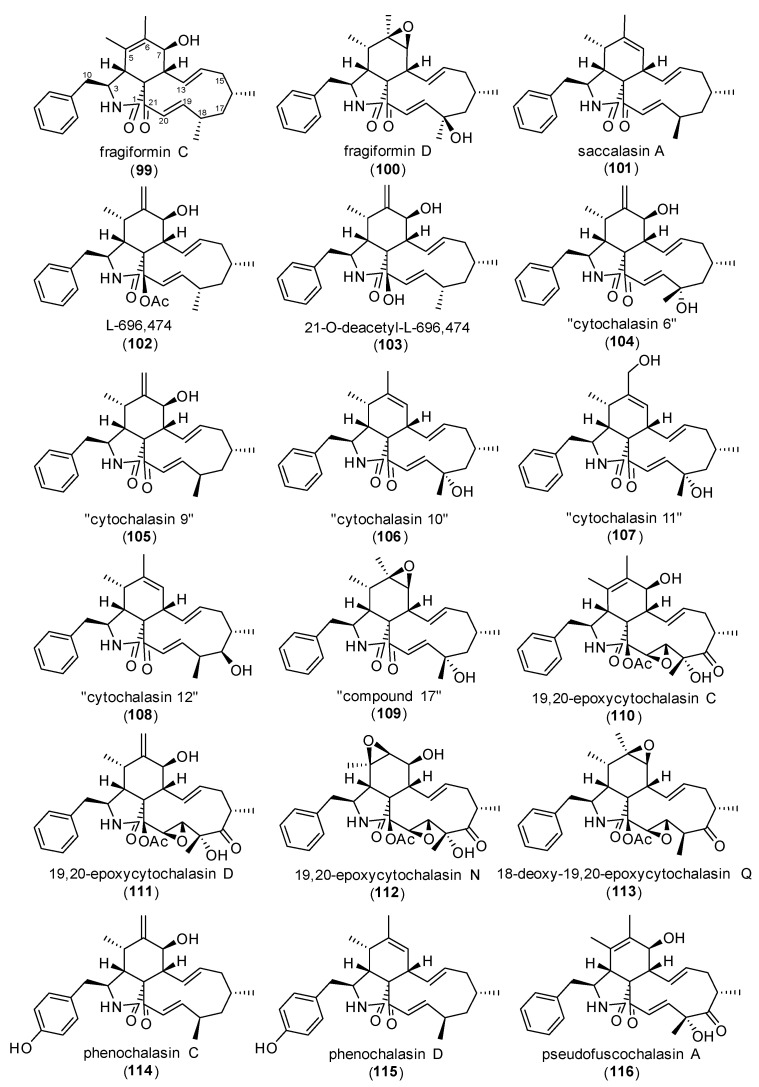
Structures of cytochalasans tested by Kretz et al. (2010) (**99**–**115**) and Lambert et al. (2021) (**116**) [76,78].

**Figure 10:**
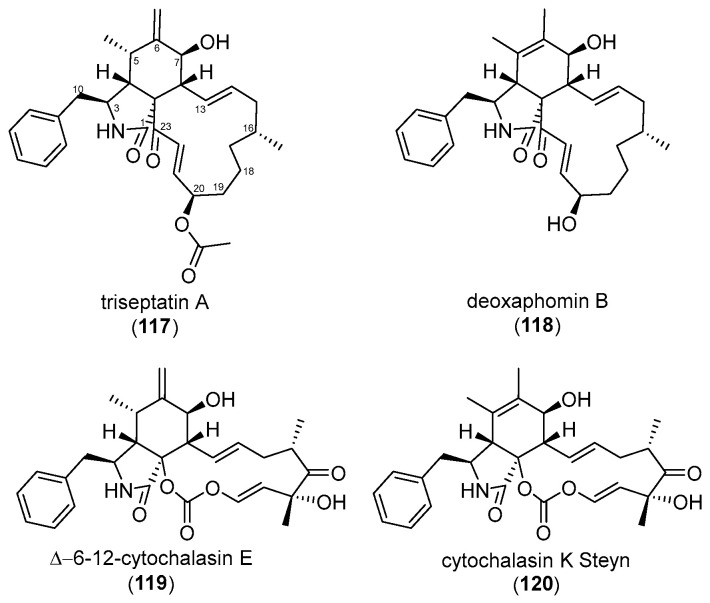
Structures of cytochalasans investigated by Garcia et al. (2022) (**117**, **118**) and Pourmoghaddam et al. (2022) (**119**, **120**) [77,79].

**Figure 11:**
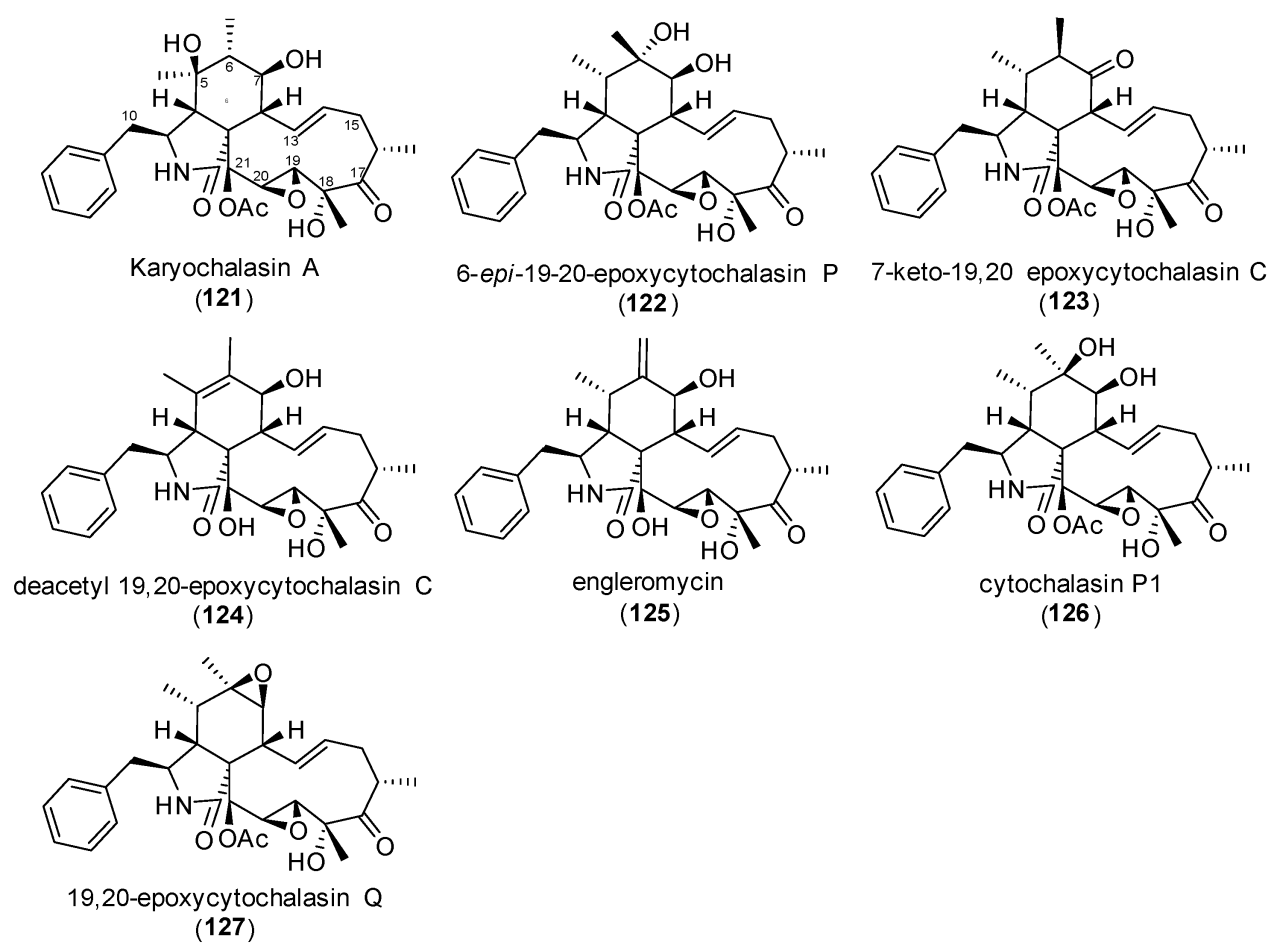
Structures of cytochalasans (**121**–**127**) tested by Lambert et al. (2023) [96].

**Figure 12:**
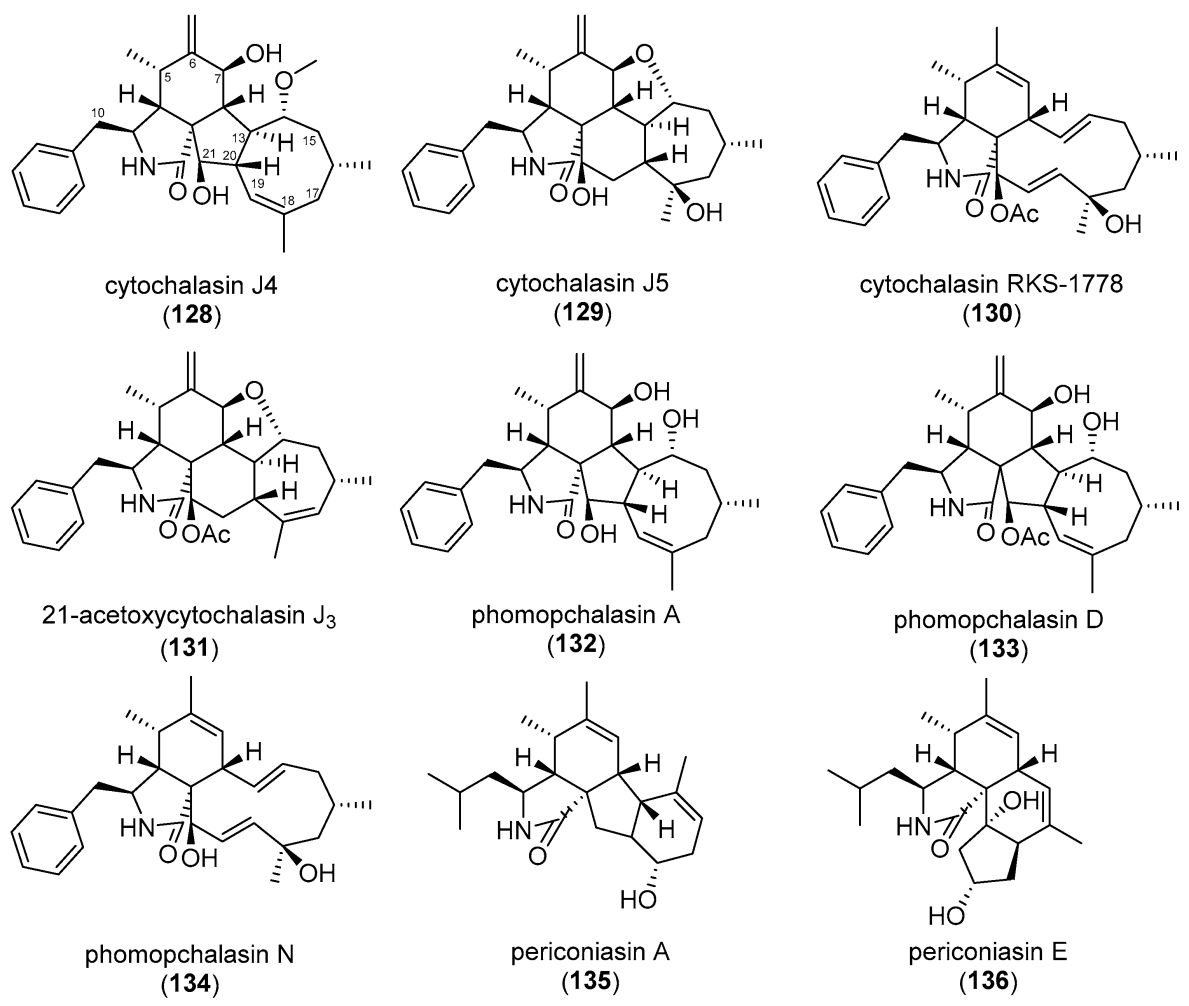
Structures of cytochalasins (**128**–**134**) analyzed by Kemkuignou et al. (2022) and periconiasin A and E (**135–136**) [97,99,100].

**Scheme 1:**
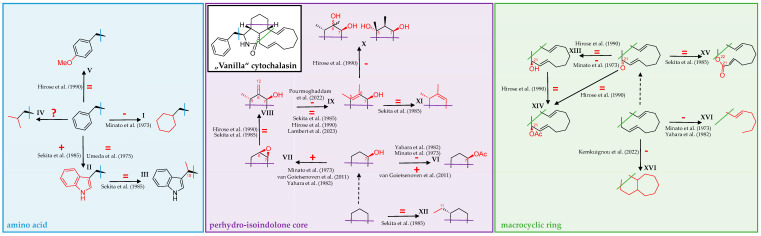
Over the past five decades, a multitude of cytochalasin derivatives has been shown to harbor differential efficacy when tested for cytotoxicity and bioactivity in functional cell biological assays towards actin-dependent structures and actin polymerization in biochemical experiments. Modifications at the incorporated amino acid (light-blue box, **left**), perhydro-isoindolone core (purple box, **middle**), and macrocyclic ring (light green box, **right**) have by and large been independently regarded from the remaining cytochalasan backbone (blue, purple, and green lines drawn into a “Vanilla” cytochalasin in the **middle top-left**). Derivatives featuring a de-aromatized phenylalanine unit were shown to elicit reduced activity (**I**). Tryptophane-derived indole-substituted compounds were reported to either increase or have no effect on bioactivity (**II**), while other non-ribosomal peptide substituents have so far not been comprehensively studied (**IV**). No effect on bioactivity was found for a methyl attached to C-10 (**III**), or a methoxy group substituted in para position at the phenyl shown in **V**. Acetylation of a C-7 hydroxy group found in the perhydro-isoindolone core was shown to either decrease or increase efficacy (**VI**), while substitution with a C-6/C-7 epoxide increased activity (**VII**). Toxicity was unchanged for a combination of C-7 hydroxy, C-6 methylene, and C-5 methyl moieties (**VIII**), while a double bond between C-5 and C-6 either had no influence or decreased efficacy compared to the former (**IX**). Epimers of further hydroxylated compounds were shown to display decreased activity (**X**). However, compounds lacking oxygen functions in the perhydro-isoindolone core did not differ in efficacy compared to oxygenated ones (**XI**). The same was reported for a methylated methyl attached to C-11 (**XII**). Hydroxylation of a keto-group at C-21 was without effect or decreased efficacy (**XIII**), while acetylation did not change activity, neither when compared to a ketone nor a hydroxy group at C-21 (**XIV**). Enlargement of the macrocyclic ring by oxygen insertion, forming a lactone, was also without effect (**XV**). Finally, ring opening and polycyclization were both reported to decrease activity (**XVI**). For conflicting reports and further structural details, see main text above. Studies cited in the scheme are as follows: Umeda et al. (1975) [55], Minato et al. (1973) [56], Sekita et al. (1985) [57], van Goietsenowen et al. (2011) [58], Yahara et al. (1982) [69], Pourmoghaddham et al. (2022) [78], Hirose et al. (1990) [85], Lambert et al. (2023) [96], and Kemkuignou et al. (2022) [97].
